# FusoBase: an online *Fusobacterium* comparative genomic analysis platform

**DOI:** 10.1093/database/bau082

**Published:** 2014-08-22

**Authors:** Mia Yang Ang, Hamed Heydari, Nick S. Jakubovics, Mahafizul Imran Mahmud, Avirup Dutta, Wei Yee Wee, Guat Jah Wong, Naresh V.R. Mutha, Shi Yang Tan, Siew Woh Choo

**Affiliations:** ^1^Genome Informatics Research Laboratory, High Impact Research Building, University of Malaya, 50603 Kuala Lumpur, Malaysia, ^2^Department of Oral Biology and Biomedical Sciences, Faculty of Dentistry, University of Malaya, 50603 Kuala Lumpur, Malaysia, ^3^Department of Software Engineering, Faculty of Computer Science and Information Technology, University of Malaya, 50603 Kuala Lumpur, Malaysia and ^4^Centre for Oral Health Research, School of Dental Sciences, Newcastle University, Framlington Place, Newcastle upon Tyne NE2 4BW, UK

## Abstract

*Fusobacterium* are anaerobic gram-negative bacteria that have been associated with a wide spectrum of human infections and diseases. As the biology of *Fusobacterium* is still not well understood, comparative genomic analysis on members of this species will provide further insights on their taxonomy, phylogeny, pathogenicity and other information that may contribute to better management of infections and diseases. To facilitate the ongoing genomic research on *Fusobacterium*, a specialized database with easy-to-use analysis tools is necessary. Here we present FusoBase, an online database providing access to genome-wide annotated sequences of *Fusobacterium* strains as well as bioinformatics tools, to support the expanding scientific community. Using our custom-developed Pairwise Genome Comparison tool, we demonstrate how differences between two user-defined genomes and how insertion of putative prophages can be identified. In addition, Pathogenomics Profiling Tool is capable of clustering predicted genes across *Fusobacterium* strains and visualizing the results in the form of a heat map with dendrogram.

**Database URL:** http://fusobacterium.um.edu.my.

## Introduction

*Fusobacterium* is a genus of gram-negative anaerobic non-sporulating bacteria in which individual cells are rod-shaped with tapered ends (1). *Fusobacterium* spp. are part of the normal flora of humans and animals. In humans, *Fusobacterium nucleatum* is widely present in the oropharynx and gastrointestinal tracts (2, 3). However, there is strong evidence that *Fusobacterium* spp. contributed to a variety of diseases. The majority of research has focused on the role of *Fusobacterium* spp. in gingivitis and periodontitis, where *F.* *nucleatum* is thought to play a role in recruiting periodontal pathogens into subgingival dental plaque. Periodontitis is a risk factor for preterm birth, and there is evidence that maternal oral strains of *F. nucleatum* are involved (4). *Fusobacterium* spp. can occasionally cause localized infections such as tonsillitis, para-tonsillar abscess or dental sepsis or invasive disease, including septicemia, abscesses of the brain, liver or lung or Lemierre’s syndrome, a septic thrombophlebitis of the internal jugular vein that is often associated with oropharyngeal infection (5, 6). *Fusobacterium* spp. are also considered major pathogens in noma (cancrum oris), a devastating facial infection that occurs in conditions of poverty and malnutrition, most commonly in sub-Saharan Africa (7). Invasive infections most commonly caused by *Fusobacterium necrophorum,* are included under the broad term of ‘invasive *F.* *necrophorum* disease’, which is a rare condition occurring to previously healthy young people, while the factors triggering the invasive process are still unclear (8). Recently, *Fusobacterium* has gained widespread attention for its association with colorectal cancer (9–11), with studies suggesting that these bacteria may have a fitness advantage in the evolving tumor microenvironment. Apart from that, *Fusobacterium* may also be associated with inflammatory bowel diseases, which is a known risk factor for colorectal cancer (12, 13).

With advances in sequencing technology, many genomes of *Fusobacterium* spp. have been sequenced by researchers around the globe (14–19). It is expected that the number of sequenced genomes will rapidly increase especially when sequencing costs further reduce. Analysis carried out by Cole and coworkers showed that the importance of comparative genomic analysis on these sequenced strains may provide further insights on their phylogeny, pathogenicity and other information that may contribute to better management of diseases caused by bacteria (20).

The collection of bacterial genomes into a single database is a new trend to analyze their genomes effectively. In recent years, many specialized genomic databases have been developed, especially for human disease pathogens. Examples of these are *Mycobacterium abscessus* genome and annotation database (MabsBase) (21), Pseudomonas genome database (22, 23), *Mycobacterium* genome polymorphism and gene function studies database (MyBASE) (24), Cyanobacteria genome database (CyanoBase) (25–27) and many other useful databases. However, there is no such specialized genomic database available for *Fusobacterium* spp. even though this genus has been the subject of much research and many *Fusobacterium* genome sequences are available because of the blooming of the next-generation sequencing era (28).

There are a number of databases, including, for instance, the microbial genome database for comparative analysis (29–31) and the integrated microbial genomes (32–35) system, which provide a wide array of microbial genomes including some *Fusobacterium* strains for comparative genomics. However, these databases do not focus on virulence factors for comparative pathogenomics. Another concern regarding most of the existing biological databases is the lack of user-friendly Web interfaces, for example, allowing real-time and fast querying and browsing of genomic data.

To facilitate the comparative analysis between strains or species of *Fusobacterium*, we have set up a user-friendly database, FusoBase, completed with genome sequences and annotation of *Fusobacterium* spp. We also aim to provide resources for whole-genome sequences and annotations and advanced bioinformatics tools specifically designed to support the expanding *Fusobacterium* research community.

## Methods and Results

### Overview of the database

Currently, FusoBase comprises 31 genome sequences from seven species of the *Fusobacterium* genus obtained and downloaded from National Center for Biotechnology Information (NCBI) Web site (http://www.ncbi.nlm.nih.gov) as shown in Table 1

**Table 1.** List of *Fusobacterium* species in FusoBase

No.	Species	Number of draft genomes	Number of complete genomes
1	*Fusobacterium gonidiaformans*	2	0
2	*Fusobacterium mortiferum*	1	0
3	*F. necrophorum*	6	0
4	*F. nucleatum*	29	4
5	*Fusobacterium periodonticum*	4	0
6	*F. ulcerans*	2	0
7	*F. varium*	1	0
8	*Fusobacterium russii*	1	0
9	*Fusobacterium sp.*	5	0

.

For consistency, all of these sequenced genomes of *Fusobacterium* genus were annotated using the Rapid Annotation using Subsystem Technology (RAST) server (36). RAST server is a well-established and automated annotation pipeline for complete or incomplete archaeal and bacterial genomes, which identifies protein-encoding genes, rRNA and tRNA genes. This pipeline is also able to produce high-quality assessment of gene functions and generate initial metabolism reconstruction. Integration of FIGfams (37) and a subsystem approach to annotation produces two classes of asserted gene functions: subsystem-based assertions, which are based on recognition of functional variants of a subsystem, and non-subsystem-based assertions that are filled in using more common approaches based on integration of evidence from a number of tools (38). The predicted open reading frame (CDS) sequences and their annotations were then downloaded from the RAST server. All the annotations including genes, RNAs and predicted protein functions of *Fusobacterium* strains were stored in our MySQL database server. The protein-coding genes were further analyzed to obtain information such as calculation of GC content (%), predicted hydrophobicity (pH), subcellular localization and molecular weight (Da) of the encoded proteins using in-house Perl script.

### FusoBase implementation

**Figure 1.**
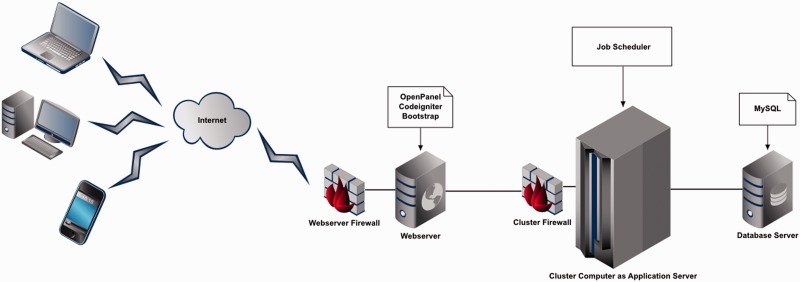
Four Tier Web application architecture of FusoBase.

The Web application was developed based on 4-tier Web application architecture (Figure 1). The Web server is built on top of Ubuntu Lucid 10.04 by OpenPanel—an open-source Web server control panel. As far as data tier is concerned, MySQL 14.12 is used as a database. A combination of PHP 5.3 and Perl 5 languages for development, Codeigniter 2.1.3 framework for Web tier and Twitter Bootstrap front-end framework for presentation layer were used.

To manage the different analytical tasks performed through BLAST, virulence factor database (VFDB) BLAST, Pairwise Genome Comparison (PGC) and Pathogenomics Profiling Tool (PathoProT), a job scheduling system was used to manage job execution and the application server utilization (39). Currently, the job queuing is of the type first in first out (FIFO). We have practiced different security services and filters, Web server and database management tools to achieve higher levels of scalability, performance, reliability and security.

Our architecture allows users with different kinds of computing devices to access and submit their jobs using the front-end provided securely by the Web server via Internet. The submitted jobs are pushed to the Cluster server, which acts as the application server, which in turn manages the jobs using a job scheduler to enable rapid processing of the jobs. In any case, whether the users interact with the Web site to submit a job or search or browse for data, the required portion of data is retrieved from the database in a fast and secure manner. This has been done by designing a highly normalized and optimized database schema.

### Browsing *Fusobacterium* strains

**Figure 2.**
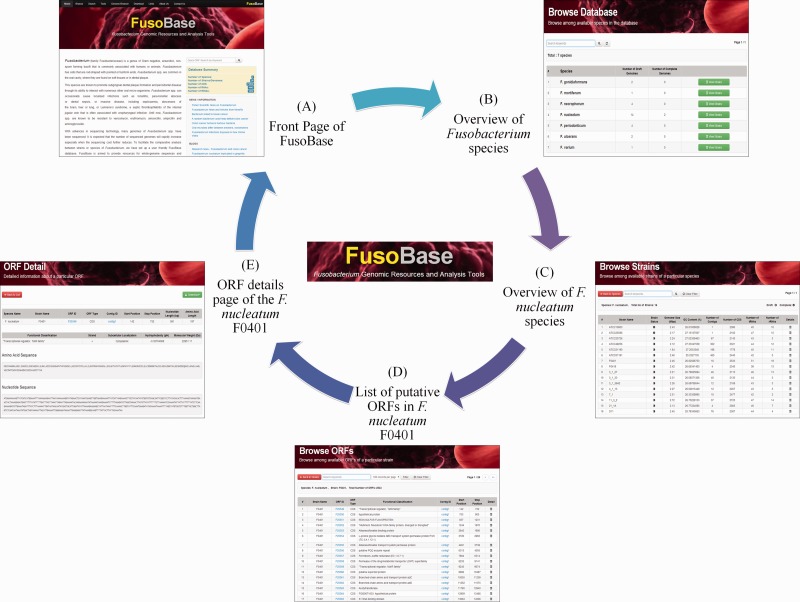
Browsing *Fusobacterium* strains. (**A**) Front page of FusoBase. (**B**) Overview of *Fusobacterium* species. (**C**) Overview of strains of* F. nucleatum* species. (**D**) List of putative ORFs in *F. nucleatum* ATCC10953. (**E**) ORF details page of the *F. nucleatum* ATCC10953.

The browse page of this database summarizes a list of seven *Fusobacterium* spp., whereby users can obtain strain-specific information such as the genome size, CDSs, number of tRNAs and rRNAs and GC content (Figure 2). Hyperlinks are provided in the details section, for strain name, ORF ID and contig ID, providing links to the Web page at NCBI (40–42), enabling the users to find more information about the strains.

The ORF list of each strain is accessible by clicking the ORF link of the desired strain. Detailed information of an ORF such as its ORF ID, type, function or subsystem classification and its start and stop positions are available with the built-in Jbrowse (43) to enable users to further visualize the ORF within a particular contig. Alternatively, users can perform a quick search for information under the database search tab by applying query filters for strain type, ORF ID or relevant keywords, without having to scroll through the full list of available strains.

### Real-time data searching feature

**Figure 3.**
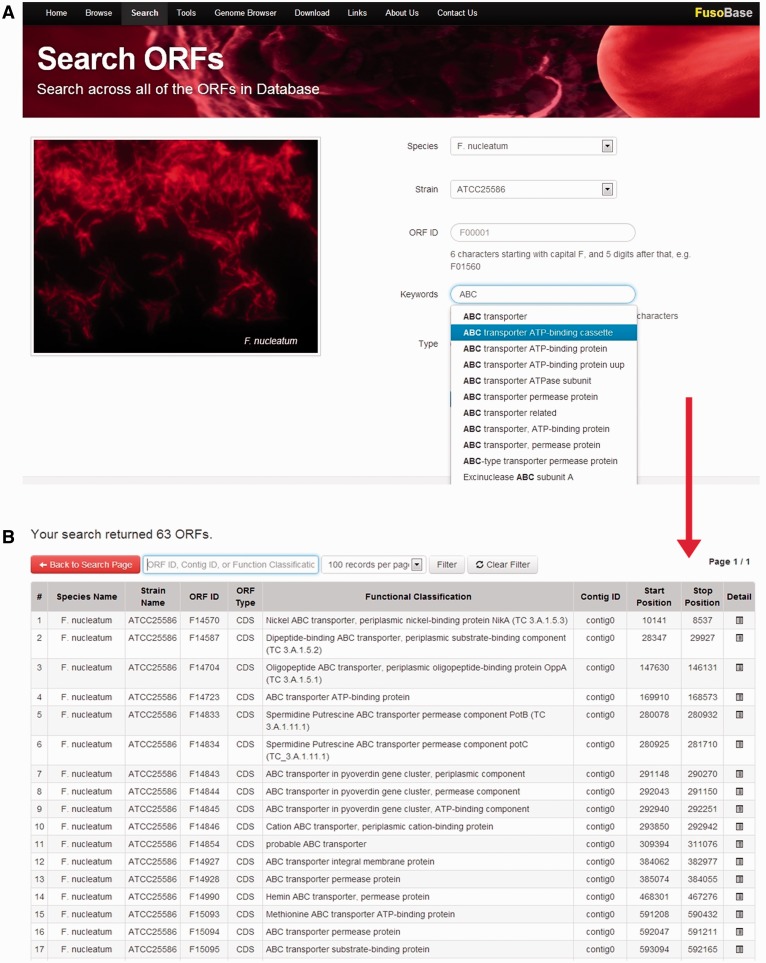
Real-time data searching system.

Annotated features in FusoBase can be obtained in different ways. We have integrated a powerful real-time AJAX-based search function in the ‘Search’ tool in FusoBase home page. The search parameters include species, strain, ORF ID, keywords of functional classification and type of sequence. Users can search the whole database by selecting a specific species name, which will display all the strains of corresponding species. From there, filtration can be done specifically according to the ORF ID, contig ID or functional classification as shown in Figure 3. The system will then retrieve the matches in real time as soon as users input the desired keywords. The search function has been designed to speed up the searching and getting the right keywords, especially when dealing with a large data set.

### Genome comparison and visualization with PGC tool

As mentioned in the previous sections, many genomes of *Fusobacterium* spp. have already been sequenced by different researchers around the globe, and many more of the newly sequenced *Fusobacterium* genomes are expected to become available soon. With an organism like *Fusobacterium*, which is so widely distributed in humans and many of its species already being associated with a variety of diseases, we still do not have a clear understanding of its biology. Genomic information from a single *Fusobacterium* genome is not enough, for giving us a clear picture of the lifestyle and extended view of the gene pool of the species. A comparative study of multiple *Fusobacterium* genomes could provide us with a highly detailed view of the relatedness and the variations between the organisms at the genetic level, giving us a better understanding about their physiology. Comparative genomic analysis of the *Fusobacterium* genomes will enable us to study the evolutionary changes among organisms, identify the orthologous genes in species, as well as genes that give each organism its unique characteristics, explore the genetic differences, e.g. indels and rearrangements between two genomes either inter- or intraspecies, evolutionary signals and potential regions associated with pathogenicity.

With that in mind, the PGC tool was developed and incorporated into FusoBase. This unique analysis tool will allow the users to compare and visualize two genomes of interest. In the Tool’s page, researchers can select the genomes of two *Fusobacterium* strains of interest and compare them with the PGC tool. Alternatively, users can upload their own genome sequence and compare with one of the strains in FusoBase through our custom submission form. One of these genomes will be considered as a reference genome, while the other one will be labeled as query genome for comparison purposes.

The PGC tool is a consolidated tool aligning whole-genome sequences using the NUCmer program of MUMmer package (44) and Circos (45) for visualization of pairwise comparison between two cross-strain/species. Right after the alignments are generated through NUCmer, PGC tool immediately parses the results to Circos, which will then generate a circular ideogram layout to show the relationship between pairs of positions, with karyotypes and links encoding the position, size and orientation of the related genomic elements. The multi-step process of this pipeline was automated using Perl scripts and the results can be visualized in just a few minutes. The results generated by the PGC tool, both the NUCmer results and the Circos plot along with a help file can be downloaded using the ‘Download’ button in the PGC result page. Supplementary Figure S1 briefly illustrates the work flow of PGC tools describing the integration of both MUMmer and Circos, generating the required input files for the PGC to function.

**Figure 4.**
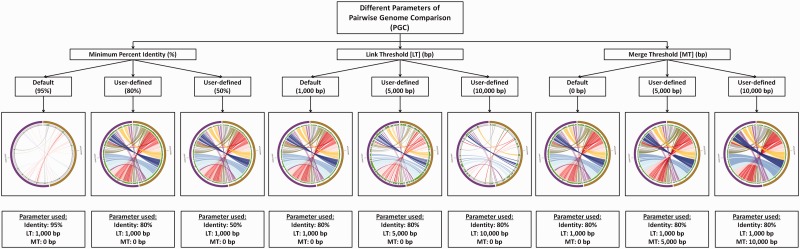
The influence of different cutoffs of minimum percent identity (%), LT and MT on the final output of PGC.

PGC allows users to configure parameters, such as (i) minimum percent genome identity (%), (ii) link threshold (LT; bp), which removes the links according to user-defined value and (iii) merge threshold (MT; bp), which allows merging of links based on user-defined value. By default, the thresholds of the PGC tool are set to be 95% minimum percent identity and 1000 bp LT. The users may change the parameter freely to get different comparative results. The influences of different parameters on the comparative results and the display of the aligned genomes are shown in Figure 4. Apart from these parameters, users can input their email addresses for the analysis result to be sent directly to their emails once the analysis is completed by the PGC pipeline.

Development of PGC is inspired by similar tools, like Circoletto (46) and RCircos (47). Designed to excel more than these tools, PGC has some distinct advantages over these online tools. Compared with Circoletto, which aligns desired sequences using BLAST (local alignment); the alignment algorithm used in PGC is based on the NUCmer (global alignment) package in MUMmer, which is more suitable for large-scale and rapid genome alignment (44, 48). Apart from that, PGC provides users with options for adjusting settings such as minimum percent genome identity (%), merging of links/ribbons according to MT and the removal of links according to the user-defined LT through the provided online form. Further, a histogram track is added in the circular layout generated by PGC, which shows the percentage of mapped regions along the genomes. This track is useful and helps users to identify putative indels and repetitive regions in the compared genomes. Supplementary Figure S2A shows how the MT works with the merging process by PGC tool, while Supplementary Figure S2B shows how the data in the histogram track is calculated.

Besides the Circoletto, RCircos is another tool with similar function to PGC, which was developed using R packages that comes with R base installation (47). The package supports Circos 2D data track plots such as scatter, line, histogram, heat map, tile, connectors, links and text labels. Each plot was implemented with a specific function, and input data for all functions were data frames, which can be objects read from text files or generated with other R pipelines. PGC has a distinct advantage over RCircos, for PGC provides a user-friendly interface and easy-to-use add-on marks, which unlike RCircos, requires no prior knowledge in programming languages in running the package.

**Figure 5.**
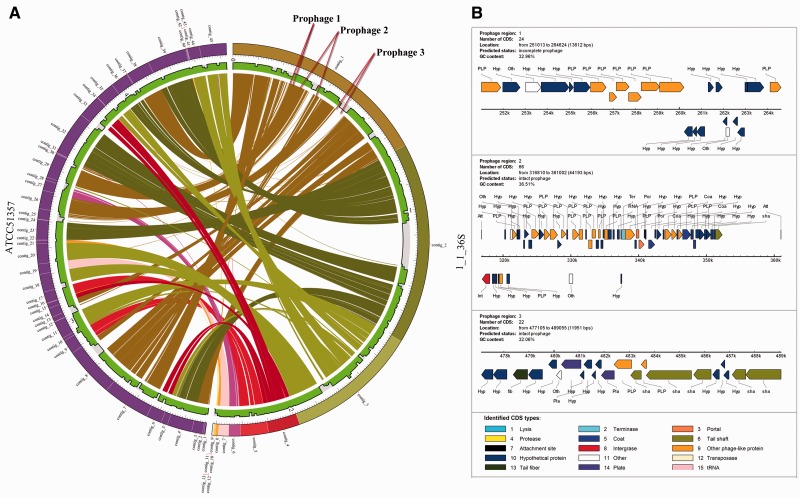
Comparison between two genomes using PGC reveals putative prophages. (**A**) Visualization of two aligned draft genomes, *F. necrophorum* 1_1_36S and *F. necrophorum* ATCC51357 revealing three insertions in the *F. necrophorum* 1_1_36S genome, which are associated with putative prophages as predicted by PHAST. (**B**) Overview of the CDSs in the putative prophages.

To demonstrate the handy feature of our PGC pipeline, we compared two draft genomes of *F.* *necrophorum* 1_1_36S and *F.* *necrophorum* ATCC51357, as displayed in Figure 5A. As shown in the Circos plot, the genome comparison revealed some genome differences between the closely related strains. For instance, we have identified a number of white gaps or regions that were not aligned to *F.* *necrophorum* ATCC51357. This prompted us to investigate further, and we wanted to find out whether there were any phage-acquired regions in these genomes. To predict the phage-acquired regions in these genomes, we used the online PHAge Search Tool (PHAST) (49). PHAST identified three prophage regions in the genome of *F.* *necrophorum* 1_1_36S as shown in Figure 5B. Prophage 1 is a putative incomplete prophage and has a genomic length of 13.6 kb and G+C content of 32.9%. Prophage 2 is an intact or complete prophage with a genomic length of 44.1 kb and G+C content of 36.5%. Prophage 3 is also an intact prophage observed in this genome with G+C content of 32.06% Figure 5B. Interestingly, when we correlated the PHAST results with the Circos plot as obtained from the analysis using our PGC tool, we found that the predicted prophage 1 was in the aligned region whereas the predicted intact prophage 2 covered a region encompassing both aligned and unaligned regions. However, the predicted intact prophage 3 is present in the white gaps or unaligned regions in the *F. necrophorum* 1_1_36S (Supplementary Table S1). Taken together, we have shown that our PGC tool is effective in showing the relatedness and differences between two *Fusobacterium* genomes.

### Identification and comparative analysis of virulence genes with PathoProT

Virulence can be defined as a quantitative measure of the pathogenicity or the likelihood of causing disease and is the means by which pathogenic organisms express their pathogenicity. Virulence factors or genes are referred to as are any of the genetic or biochemical or structural features that enable the pathogenic organism to establish itself on or within a host and increase its chances of causing disease. These factors can be broadly divided into two major modes of pathogenesis, one of which is toxigenesis (bacterial toxins both exotoxins and endotoxins) and the other invasiveness, which includes cell surface proteins mediating bacterial attachment, extracellular substances that facilitate invasion (invasins), cell surface carbohydrates and proteins that protect a bacterium to bypass or overcome host defence mechanisms and also hydrolytic enzymes that may contribute to the pathogenicity of the bacterium (50–52). The acquisition of individual virulence factors may convert nonpathogenic bacteria into pathogens (53). To identify genes that express virulence factors, PathoProT was developed. PathoProT is a pipeline developed using in-house Perl and R scripts. All the virulence factors from different pathogenic organisms, which have been experimentally verified, are curated in the VFDB. These virulence factors retrieved from VFDB (VFDB Version 2012 containing 19 775 proteins) for PathoProT are considered as the ‘known virulence factors’. In the PathoProT pipeline, the orthologs of these known virulence factors are identified in the *Fusobacterium* genomes present in the FusoBase by BLAST search with the default parameters of 50% sequence identity and 50% sequence completeness. However, these default parameters for the BLAST search can be changed by the user depending on their desired levels of stringency. The orthologs thus identified in the *Fusobacterium* genomes are the ‘predicted virulence genes’. The predictions of these orthologs are done through the use of the well-established BLAST (Stand-alone) tool of NCBI (39, 54–56), which is downloaded from NCBI and embedded in the pipeline of PathoProT. The Perl script handles the early process, where it is programmed to filter the BLAST results generated from BLAST search against VFDB and selects only the user-desired strains. The selected results are then structured into a data matrix form, in which the correlation between strain/genomes and the virulence genes are displayed. The Perl scripts then manipulate the data frame by assigning 1 and 0 values for ‘presence’ and ‘absence’ of virulence genes, respectively. The data frame is then converted into a data matrix, and the initial result will be passed to the R script, generating hierarchical clustering of the virulence genes and producing a heat map of multiple virulence gene profiles. Supplementary Figure S3 shows the flow chart of PathoProT, briefly describing the pipeline, which integrates both Perl script and R script, and the processes before generating the output file.

**Figure 6.**
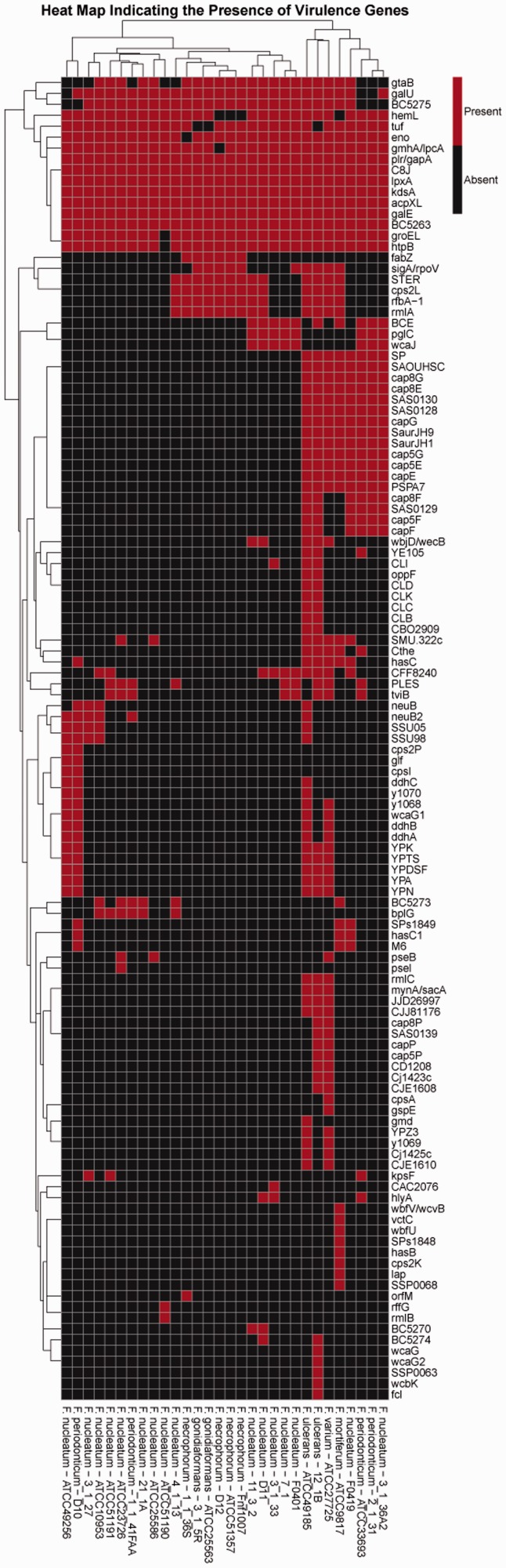
The heat map generated using PathoProT, showing the cluster of *Fusobacterium* strains based on their virulence gene profiles. The threshold used in this analysis was 50% sequence identity and 50% sequence completeness.

On testing, PathoProT predicted all the known virulence genes based on the pathogenomic composition of the genomes by clustering predicted virulence genes across different selected strains. The final output is generated in the form of a heat map, combined with side-by-side dendrograms (Figure 6) giving a bird’s eye view of the distribution of the virulence genes across *Fusobacterium* spp. The combination of heat map and dendrogram shows a neat overview of the clustered strains, which have closely related sets of virulence genes present in each group of clustering. In addition, the strains are sorted according to the level of similarities across the strains and virulence genes. Seven virulence genes, *plr/gapA*, *C8J*, *lpxA*, *kdsA*, *acpXL*, *galE* and *BC5263*, are found to be widely distributed across all the *Fusobacterium* spp., whereas other virulence genes are less widely distributed. From the generated heat map, we can clearly identify that *Fusobacterium ulcerans* has the most virulence genes, making it potentially the most pathogenic species among all *Fusobacterium* strains. To enable the user to further analyze the results generated from PathoProT, download options for the BLAST alignment result (.txt), overview of virulence genes (.txt) and the Heat Map (.pdf) are provided.

Identification of these virulence traits of *Fusobacterium* spp. are often attributable to the use of comparative pathogenomics approaches, which improve comprehension of the virulence nature and evolution, allowing new therapeutic and preventive solutions to be all the more possible (57, 58). As demonstrated, PathoProT can be used to identify virulence genes in *Fusobacterium* strains by sequence homology. Moreover, comparative pathogenomics analysis can be easily performed to compare strains/groups of strains e.g. nonpathogenic strains versus pathogenic strains, giving better insights into the biology, evolution and virulence of the *Fusobacterium* strains of interest.

### Other tools

BLAST is the foundation tool for providing alignment information and findings for genomic differences through sequence similarity search is incorporated into the FusoBase. BLAST is an algorithm to compare query sequence with a library of databases of sequence, and identify library sequences that resemble the query sequence above a certain threshold value (39, 54). In FusoBase, two different BLAST searches are provided. A standard BLAST interface, with different BLAST search options: BLASTN (CDS nucleotide sequences), BLASTN (*Fusobacterium* whole-genome sequences), BLASTP and BLASTX (Supplementary Figure S4). Besides the standard BLAST searches, there is also a VFDB BLAST (50–52). The incorporation of VFDB BLAST into FusoBase enables users to identify whether their genes of interest are potential virulence genes based on sequence homology.

Another tool implemented in FusoBase is JBrowse, a fast and modern JavaScript-based genome browser (43). JBrowse can be used to navigate genome annotations over the Web and helps preserve the user’s sense of location by avoiding discontinuous transitions, offering smooth animated panning, zooming, navigation and track selection. It uses the information of the start and end location, predicted function and subsystem information to display the strand and provides information on it. JBrowse is integrated into FusoBase to ensure high-speed visualization of selected *Fusobacterium* strains, which refreshes and reloads the Web page as soon as user changes the input strains. The genome browser provides three user-defined options (in the dropdown menu), for the selection of species, strains and contig. Integration of JBrowse inside FusoBase enables visualization of contigs, DNA sequences, RNA sequences and genome annotation results (Supplementary Figure S5).

### Protein subcellular localization analysis

Subcellular localization prediction of predicted proteins was analyzed using the latest PSORTb version 3.0, which categorizes the proteins with high-confidence subcellular scores (59, 60). Preliminary analysis revealed that these *Fusobacterium* proteins can be categorized into the following five categories of, cytoplasmic, cytoplasmic membrane, extracellular, outer membrane and periplasmic (Supplementary Figure S6). Based on the analysis performed, more than half of these *Fusobacterium* proteins (55.9%) were predicted to be localized in cytoplasm. Furthermore, a high percentage of these proteins (18.4%) were predicted to be localized in cytoplasmic membrane.

### Pan-genome analysis on *Fusobacterium* genus

The *Fusobacterium* pan-genome analysis was performed using the genomic and the proteomic data from the FusoBase. The analyses included 30 strains comprising seven different *Fusobacterium* spp. The genes and proteins sequences for all these strains were downloaded from FusoBase and used as the input for the pan-genome analysis using PGAP (Pan-Genomes Analysis Pipeline) version 1.1 (61).

**Figure 7.**
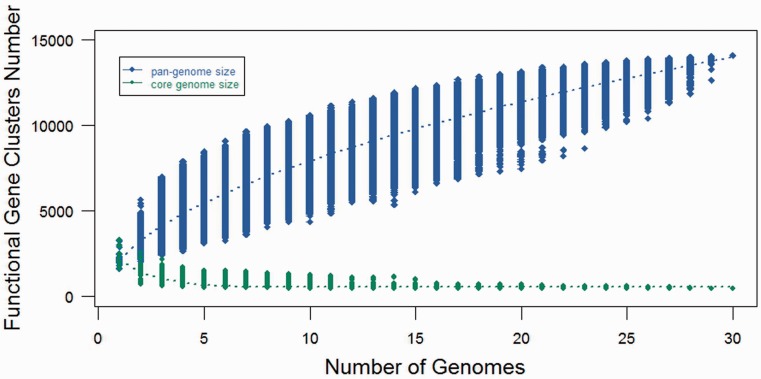
Pan- and core-genomes size prediction for *Fusobacterium*. The blue curve denotes the pan-genome size, while the red curve represents the core-genome size. The dot represents every genome comparison with different combination and the median values have been used to draw the curve.

After clustering all the functional genes from these 30 strains, we identified that of the 14 101 gene clusters, there were only 462 genes that could be considered as core genes, which were shared by all the 30 strains, whereas 13 639 genes (accessory genes) were not shared by all the strains. The low number of core genes suggests that genus *Fusobacterium* contains a diverse genome structure. After obtaining the number of core and accessory genes, we tried to predict the pan and core genome size of the genus through an extrapolation method. Gene clusters and core clusters for N genomes were calculated, where N is the number of *Fusobacterium* genomes (*N* = 1, 2, 3, … 30). For each N genome, the pan-genome size and core genome for each of the permutations of genome comparisons was predicted. The curve for the pan-genome size can be represented by the following mathematical function of Y = 2735.2287 X^0.5 ^+ 544.4458 (R^2 ^= 0.99). In the function, Y represents pan-genome size, while X represents number of sequenced genomes. Not surprisingly, the result showed that *Fusobacterium* genus possesses an open pan-genome and the pan-genome size increased rapidly when the number of genomes increased (Figure 7). This suggested that *Fusobacterium* is continuously gaining new genes and actively evolving throughout the time frame. This also indicates that *Fusobacterium* is capable of acquiring new phenotypes through evolution (62).

**Figure 8.**
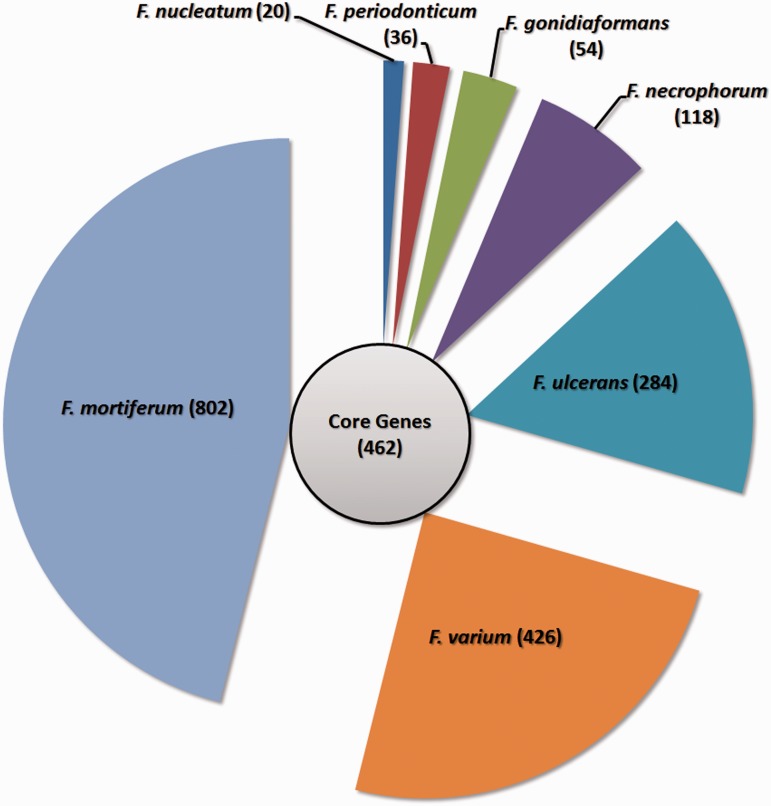
Gene distributions for the *Fusobacterium* genus. The circle in the middle denotes the core gene cluster for the *Fusobacterium* genus, while the seven segments surrounding the circle represent the number of specific genes for each *Fusobacterium* species.

From the accessory genes, we also identified the subgroup specific genes as well as the gene clusters specific to each species as shown in Figure 8. The result shows that *F. mortiferum*, *F. varium* and *F.* *ulcerans* are the three species with the highest number of unique genes. However, there is only one strain included for *F.* *mortifererum* and *F.* *varium* in the analysis, and thus, some of the genes may be strain-specific genes and not species-specific genes. Identification of these species-specific genes may be useful, as these genes might become gene markers for each of the *Fusobacterium* spp.

**Figure 9.**
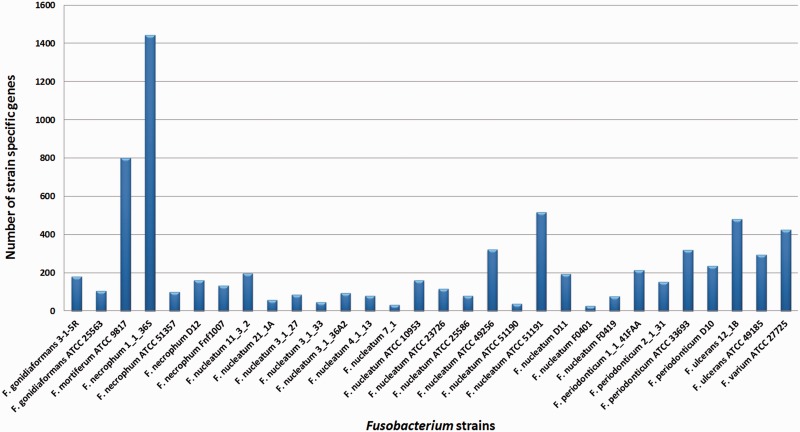
Strain-specific genes for the *Fusobacterium* genomes. *Fusobacterium necrophum* 1_1_36S has 1443 strain-specific gene clusters, which is the highest and *F. nucleatum* F0401 has the lowest, which is 27 gene clusters.

Apart from the species-specific genes, we have also identified 7171 strain-specific gene clusters, a number that is more than half of the pan-genome size. The high number of strain-specific genes suggests that *Fusobacterium* genome exhibits extreme levels of evolutionary plasticity. The distribution of strain-specific genes for the 30 strains of *Fusobacterium* is shown in Figure 9.

The continuously evolving nature of the *Fusobacterium* genomes is clearly evident from the above study. As the pan-genome study indicated, *Fusobacterium* genomes have an open pan-genome, suggesting that with the availability of new *Fusobacterium* genomes, we are likely to see an expansion in the number of new *Fusobacterium* genes. With the availability of constantly updated database of *Fusobacterium* genomes and its analytical tools, FusoBase would provide a great platform for the researchers to probe into the continuously evolving genus of *Fusobacterium*.

## Discussion

We constructed FusoBase, which stores all genome sequences and genomic information of *Fusobacterium* strains. These data can be downloaded, browsed and searched with our real-time Ajax searching system. There is also a file transfer protocol (FTP) download option, which will allow the user to batch download the sequence and annotation files. Moreover, the *Fusobacterium* research community can browse these huge genome data using the modern JBrowse. We have also provided PGC tool, which allows the research community to perform comparative analysis of two genomes of interest and visualize the comparison results. Meanwhile, our PathoProT pipeline allows user to identify potential virulence genes that are present in *Fusobacterium* genus through the comparison of sequence homology.

To provide the most accurate and detailed information about *Fusobacterium* genus, FusoBase will be maintained and updated regularly. At the moment, FusoBase comprises seven species of *Fusobacterium* encompassing 30 strains. From the genomes, we collected 71 030 CDSs, 1662 RNAs and 1436 tRNAs. Apart from that, more analysis tools and *Fusobacterium* genomic data will be added into FusoBase in the near future.

### Future directions

We are already in the process of adding new *Fusobacterium* genomes to the FusoBase and will be updating the database with new genomes of *Fusobacterium* as and when they are available. At this moment, the database is optimized for whole-genome analysis. However, we would love to incorporate other data types as well in future. We are working on it, and we also encourage other researchers or research groups to email us at girg@um.edu.my if they wish to share their annotations, opinions and curated data with us. Suggestions for improving this database and requests for additional functions are important to us and are most welcome, as it will help us make this database a comprehensive one.

### Availability and system requirements

FusoBase is available online at http://fusobacterium.um.edu.my. All the sequences and annotations described in this article could be viewed and downloaded from the Web site. FusoBase is best viewed by Internet Explorer 8.x or higher, Mozilla Firefox® 10.x or higher, Safari 5.1 or higher, Chrome 18 or higher and any other equivalent browser software. If your browser is older, you may have trouble viewing many of our Web site features properly. This Web site is best viewed at a screen resolution of 1024 × 768 pixels or higher.

## Supplementary Data

Supplementary data are available at *Database* Online.

Supplementary Data
