# Supplementary material for: FusoBase: an online Fusobacterium comparative genomic analysis platform
Source: Database (Oxford). 2014 Aug 22;2014:bau082. doi: 10.1093/database/bau082 (PMC4141642; doi:10.1093/database/bau082)
Supplement: Supplementary Data [file supp_2014_bau082_index.html]

Supplementary Data 

# FusoBase: an online *Fusobacterium* comparative genomic analysis platform

## Supplementary Data

files

**Files in this Data Supplement:**

- Supplementary Data - zip file
