# Supplementary figures and images for: FusoBase: an online Fusobacterium comparative genomic analysis platform
Source: Database (Oxford). 2014 Aug 22;2014:bau082. doi: 10.1093/database/bau082 (PMC4141642; doi:10.1093/database/bau082)

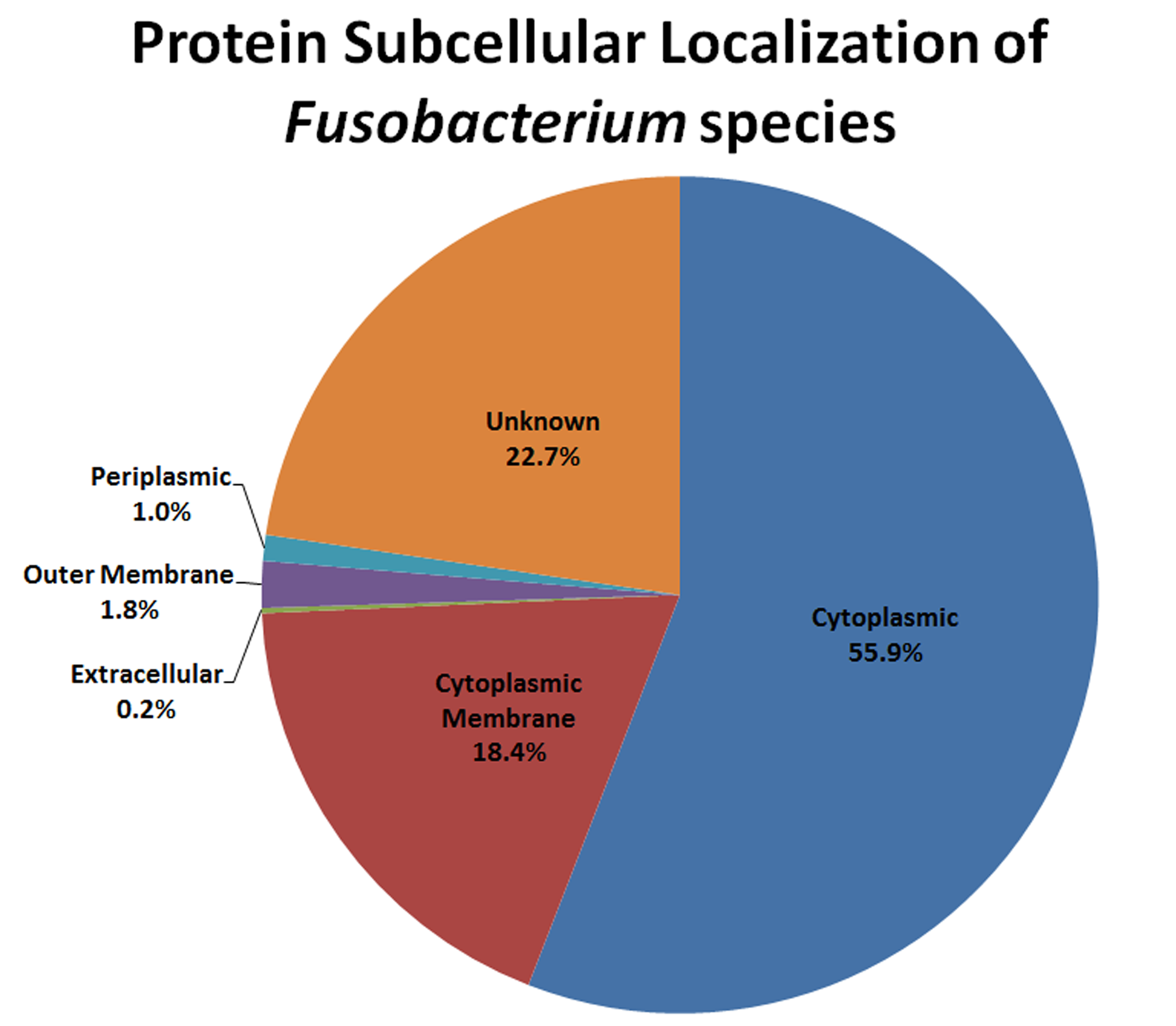

Supplement: Supplementary Data [file supp_bau082_suppl_data.zip › Supplementary_Figure_6.tif]

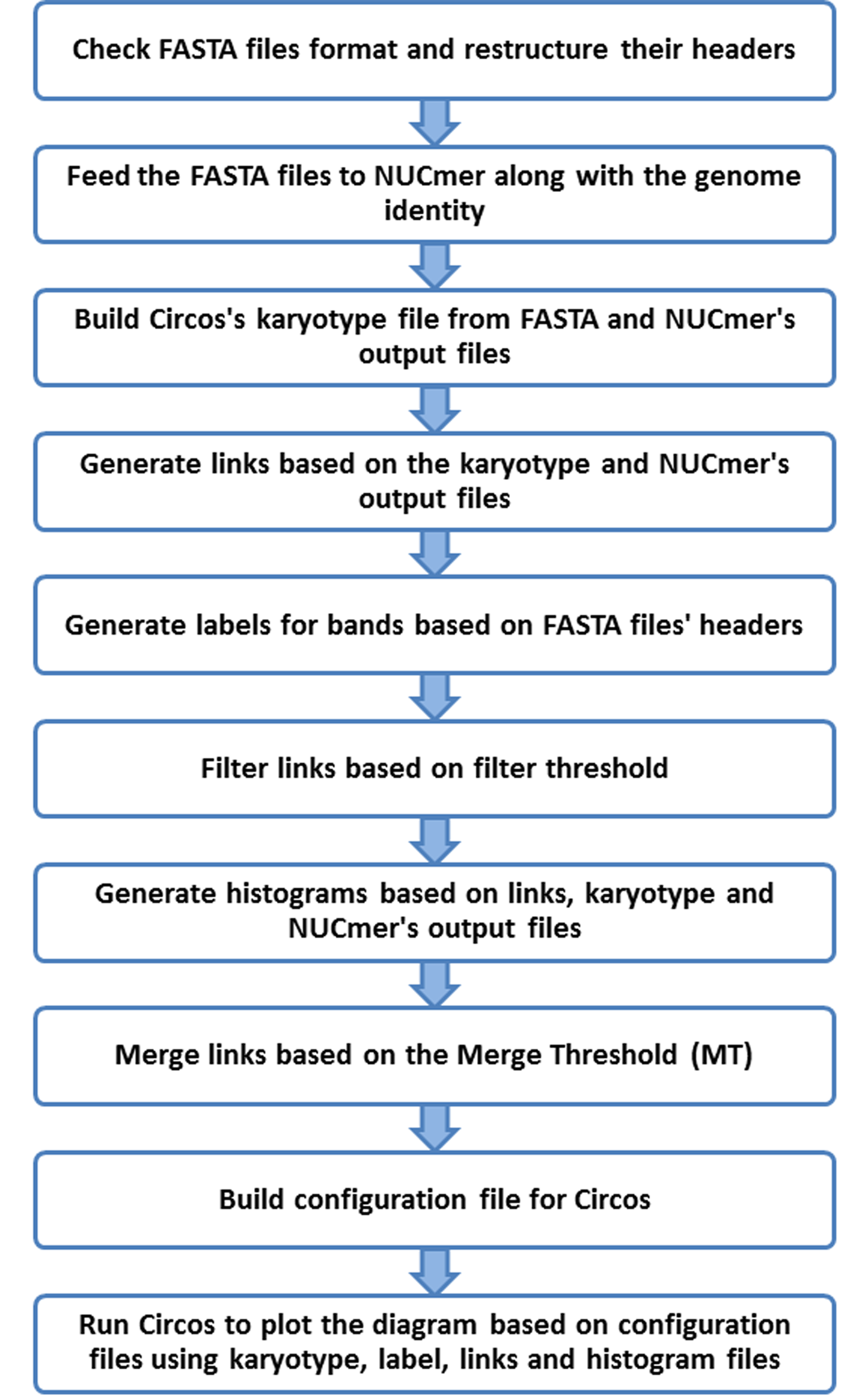

Supplement: Supplementary Data [file supp_bau082_suppl_data.zip › Supplementary_Figure_1.tif]

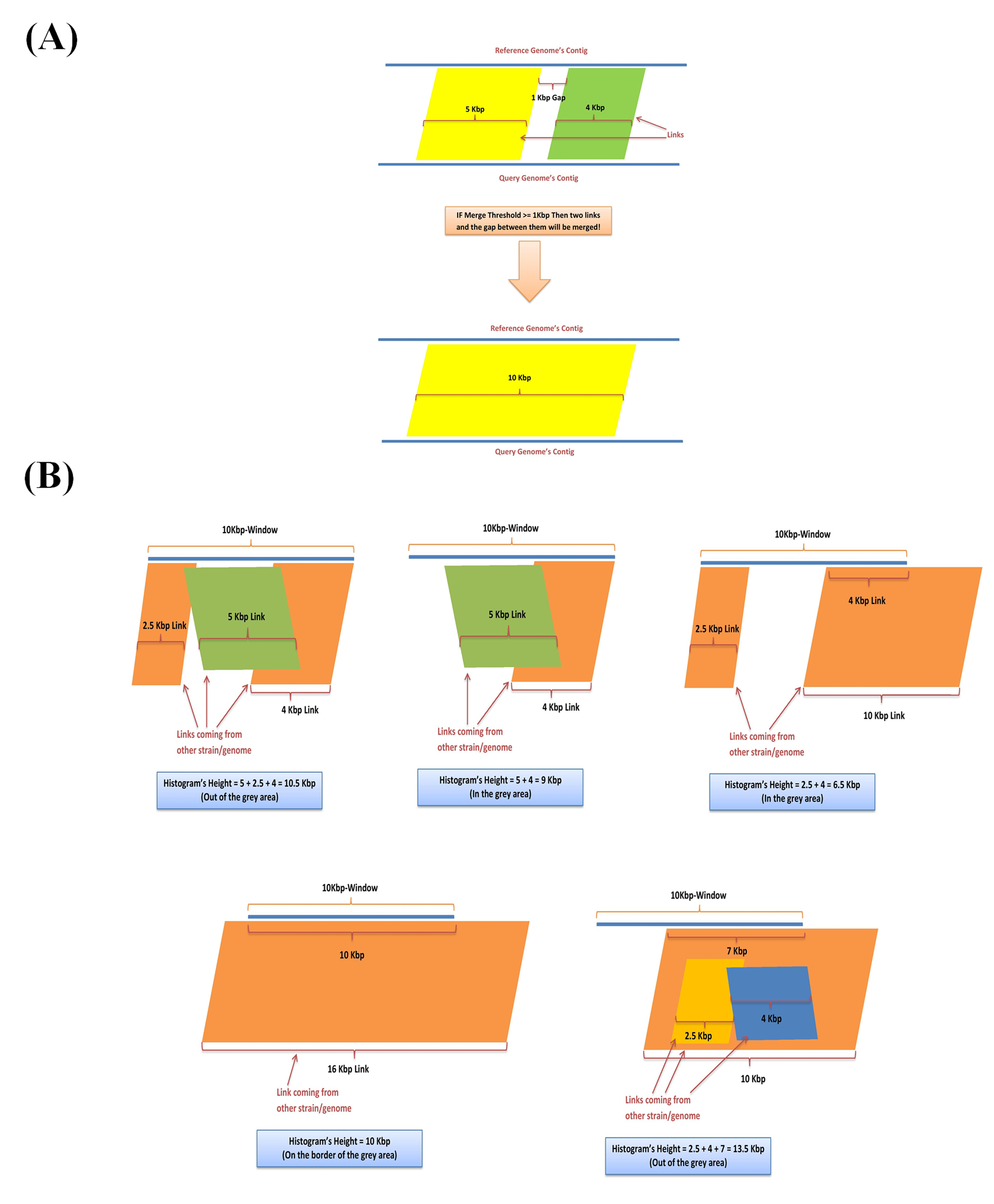

Supplement: Supplementary Data [file supp_bau082_suppl_data.zip › Supplementary_Figure_2.tif]

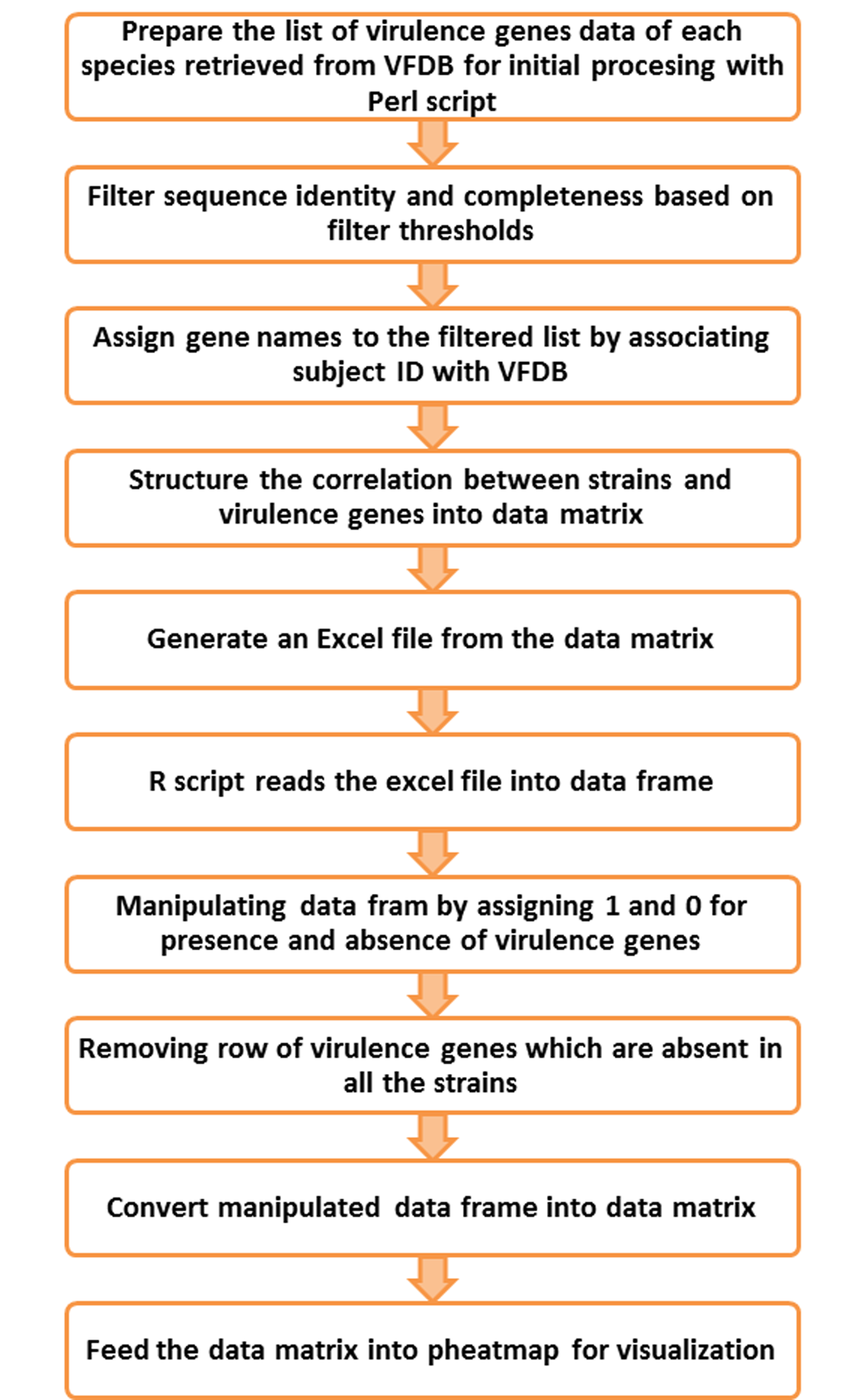

Supplement: Supplementary Data [file supp_bau082_suppl_data.zip › Supplementary_Figure_3.tif]

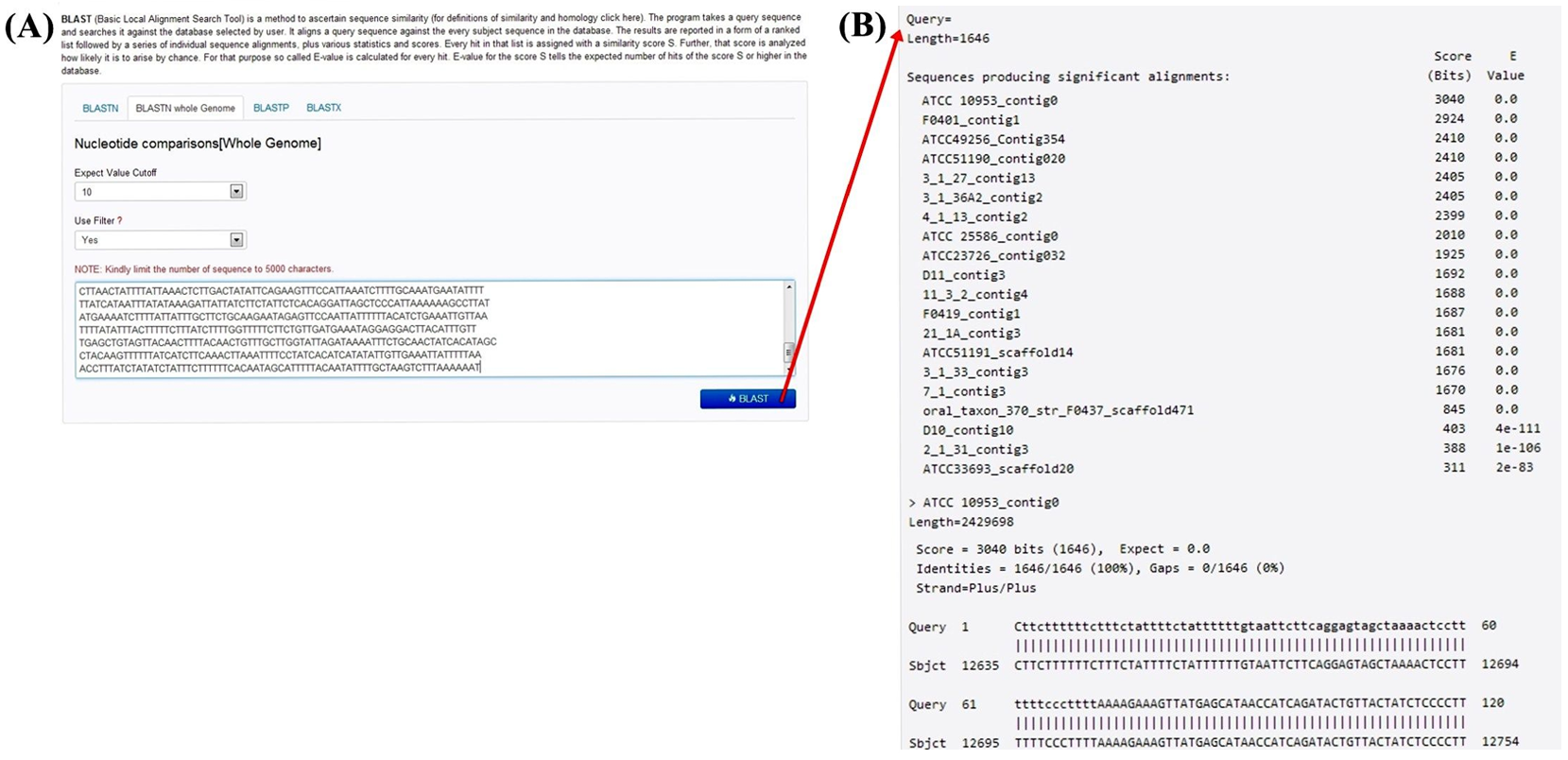

Supplement: Supplementary Data [file supp_bau082_suppl_data.zip › Supplementary_Figure_4.tif]

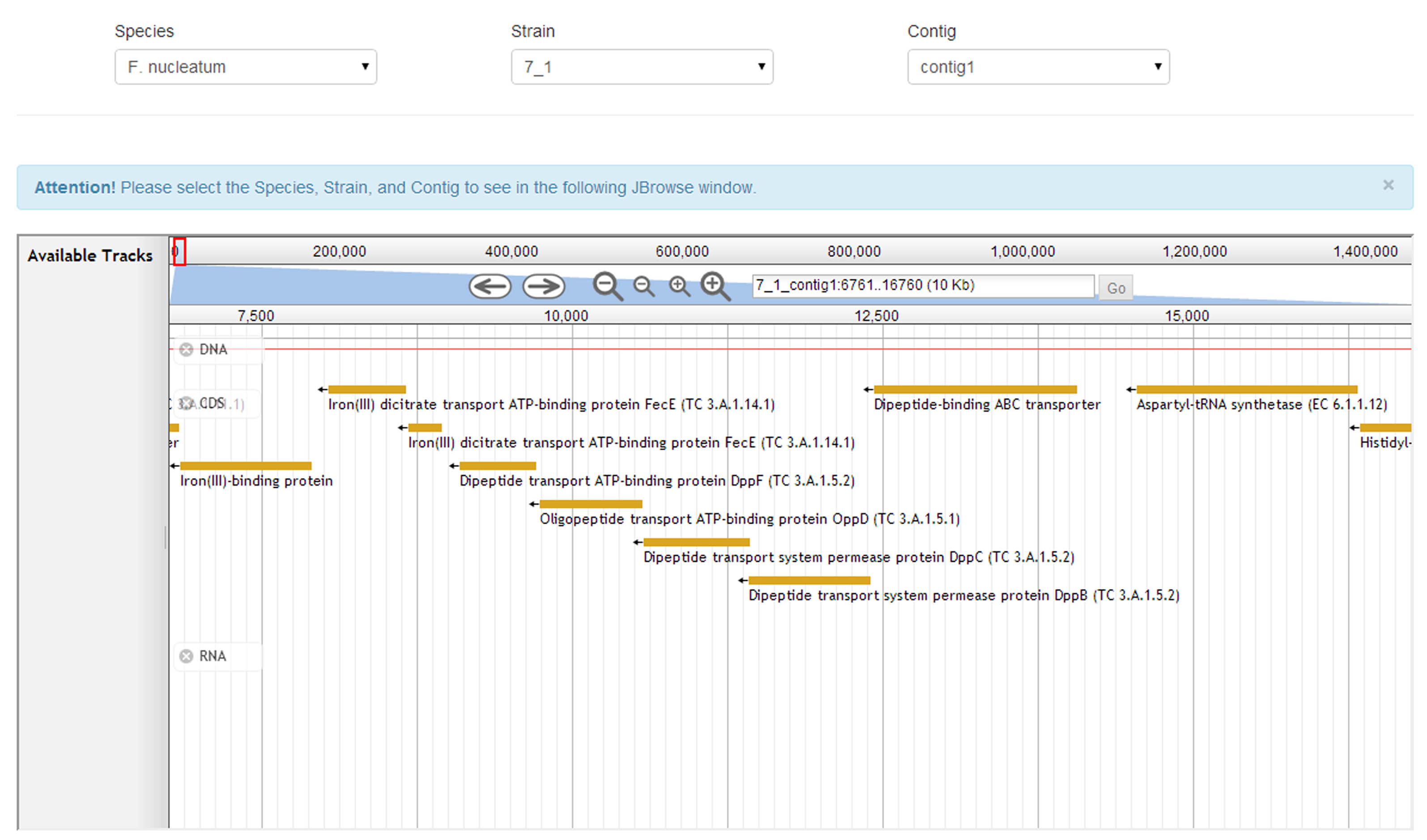

Supplement: Supplementary Data [file supp_bau082_suppl_data.zip › Supplementary_Figure_5.tif]
