# Supplementary material for: FusoBase: an online Fusobacterium comparative genomic analysis platform
Source: Database (Oxford). 2014 Aug 22;2014:bau082. doi: 10.1093/database/bau082 (PMC4141642; doi:10.1093/database/bau082)
Supplement: Supplementary Data [file supp_bau082_suppl_data.zip › Supplementary_Table_1.doc]

**Supplementary Table 1. Pairwise genome comparison between *F. necrophorum* 1_1_36S and *F. necrophorum* ATCC51357 using nucmer**

| Reference Start [S1] | Reference End [E1] | Query Start [S2] | Query End [E2] | Reference Alignment Length (bp) [LEN 1] | Query Alignment Length (bp) [LEN 2] | Percentage Identity (%) [% IDY] | Reference Tag Length (bp) [LEN R] | Quey Tag Length (bp) [LEN Q] | Reference Coverage (%) [COV R] | Query Reference (%) [COV Q] | Reference Tag [TAG R] | Query Tag [TAG Q] |
| --- | --- | --- | --- | --- | --- | --- | --- | --- | --- | --- | --- | --- |
| 1 | 316 | 316 | 1 | 316 | 316 | 100 | 761358 | 1596 | 0.04 | 19.8 | contig_1 | contig_16 |
| 343 | 84625 | 87176 | 2775 | 84283 | 84402 | 99.09 | 761358 | 87176 | 11.07 | 96.82 | contig_1 | contig_38 |
| 43382 | 43463 | 1593 | 1512 | 82 | 82 | 98.78 | 761358 | 69111 | 0.01 | 0.12 | contig_1 | contig_35 |
| 84258 | 87499 | 3243 | 1 | 3242 | 3243 | 98 | 761358 | 4708 | 0.43 | 68.88 | contig_1 | contig_1 |
| 87500 | 90585 | 1 | 3089 | 3086 | 3089 | 97.83 | 761358 | 173550 | 0.41 | 1.78 | contig_1 | contig_7 |
| 87502 | 87691 | 4706 | 4516 | 190 | 191 | 95.81 | 761358 | 4708 | 0.02 | 4.06 | contig_1 | contig_1 |
| 91827 | 92148 | 3075 | 3396 | 322 | 322 | 99.07 | 761358 | 173550 | 0.04 | 0.19 | contig_1 | contig_7 |
| 92149 | 92340 | 65350 | 65546 | 192 | 197 | 96.95 | 761358 | 65669 | 0.03 | 0.3 | contig_1 | contig_11 |
| 93532 | 93669 | 1474 | 1598 | 138 | 125 | 89.13 | 761358 | 69111 | 0.02 | 0.18 | contig_1 | contig_35 |
| 93568 | 93664 | 43952 | 44048 | 97 | 97 | 97.94 | 761358 | 87176 | 0.01 | 0.11 | contig_1 | contig_38 |
| 93667 | 93775 | 43952 | 44061 | 109 | 110 | 95.45 | 761358 | 87176 | 0.01 | 0.13 | contig_1 | contig_38 |
| 93682 | 93763 | 1512 | 1593 | 82 | 82 | 98.78 | 761358 | 69111 | 0.01 | 0.12 | contig_1 | contig_35 |
| 93763 | 153811 | 3388 | 63510 | 60049 | 60123 | 98.78 | 761358 | 173550 | 7.89 | 34.64 | contig_1 | contig_7 |
| 154158 | 160739 | 66509 | 73096 | 6582 | 6588 | 99.32 | 761358 | 173550 | 0.86 | 3.8 | contig_1 | contig_7 |
| 159394 | 159501 | 132269 | 132376 | 108 | 108 | 96.3 | 761358 | 142918 | 0.01 | 0.08 | contig_1 | contig_39 |
| 160815 | 162972 | 73674 | 75837 | 2158 | 2164 | 98.01 | 761358 | 173550 | 0.28 | 1.25 | contig_1 | contig_7 |
| 162966 | 163089 | 59878 | 59755 | 124 | 124 | 91.13 | 761358 | 69111 | 0.02 | 0.18 | contig_1 | contig_35 |
| 162970 | 163105 | 1596 | 1474 | 136 | 123 | 90.44 | 761358 | 69111 | 0.02 | 0.18 | contig_1 | contig_35 |
| 164903 | 166157 | 89892 | 91149 | 1255 | 1258 | 99.28 | 761358 | 173550 | 0.16 | 0.72 | contig_1 | contig_7 |
| 166579 | 167758 | 5776 | 6983 | 1180 | 1208 | 89.17 | 761358 | 38941 | 0.15 | 3.1 | contig_1 | contig_25 |
| 174874 | 203615 | 91289 | 120061 | 28742 | 28773 | 98.92 | 761358 | 173550 | 3.78 | 16.58 | contig_1 | contig_7 |
| 206633 | 207485 | 123144 | 123996 | 853 | 853 | 90.55 | 761358 | 173550 | 0.11 | 0.49 | contig_1 | contig_7 |
| 208201 | 210606 | 124736 | 127140 | 2406 | 2405 | 99.34 | 761358 | 173550 | 0.32 | 1.39 | contig_1 | contig_7 |
| 210603 | 210910 | 65345 | 65653 | 308 | 309 | 99.03 | 761358 | 65669 | 0.04 | 0.47 | contig_1 | contig_11 |
| 211691 | 211824 | 1474 | 1593 | 134 | 120 | 88.81 | 761358 | 69111 | 0.02 | 0.17 | contig_1 | contig_35 |
| 211727 | 211824 | 43952 | 44048 | 98 | 97 | 96.94 | 761358 | 87176 | 0.01 | 0.11 | contig_1 | contig_38 |
| 211825 | 222534 | 127133 | 137851 | 10710 | 10719 | 98.98 | 761358 | 173550 | 1.41 | 6.18 | contig_1 | contig_7 |
| 222535 | 222846 | 65350 | 65663 | 312 | 314 | 98.73 | 761358 | 65669 | 0.04 | 0.48 | contig_1 | contig_11 |
| 223414 | 223545 | 1474 | 1593 | 132 | 120 | 90.15 | 761358 | 69111 | 0.02 | 0.17 | contig_1 | contig_35 |
| 223452 | 223552 | 43955 | 44055 | 101 | 101 | 96.04 | 761358 | 87176 | 0.01 | 0.12 | contig_1 | contig_38 |
| 223546 | 223637 | 961 | 870 | 92 | 92 | 89.25 | 761358 | 3165 | 0.01 | 2.91 | contig_1 | contig_22 |
| 223546 | 224428 | 2094 | 2976 | 883 | 883 | 91.28 | 761358 | 3165 | 0.12 | 27.9 | contig_1 | contig_22 |
| 224756 | 227101 | 91951 | 89604 | 2346 | 2348 | 99.02 | 761358 | 91951 | 0.31 | 2.55 | contig_1 | contig_29 |
| 227258 | 228973 | 89448 | 87731 | 1716 | 1718 | 96.16 | 761358 | 91951 | 0.23 | 1.87 | contig_1 | contig_29 |
| 228977 | 229176 | 86544 | 86358 | 200 | 187 | 88.5 | 761358 | 91951 | 0.03 | 0.2 | contig_1 | contig_29 |
| 233957 | 238549 | 86244 | 81636 | 4593 | 4609 | 97.57 | 761358 | 91951 | 0.6 | 5.01 | contig_1 | contig_29 |
| 242421 | 242767 | 79608 | 79271 | 347 | 338 | 86.74 | 761358 | 91951 | 0.05 | 0.37 | contig_1 | contig_29 |
| 242496 | 242838 | 78580 | 78238 | 343 | 343 | 83.72 | 761358 | 91951 | 0.05 | 0.37 | contig_1 | contig_29 |
| 243627 | 244677 | 75990 | 74939 | 1051 | 1052 | 95.26 | 761358 | 91951 | 0.14 | 1.14 | contig_1 | contig_29 |
| 246491 | 318156 | 74739 | 3001 | 71666 | 71739 | 98.66 | 761358 | 91951 | 9.41 | 78.02 | contig_1 | contig_29 |
| 321038 | 322147 | 37930 | 36824 | 1110 | 1107 | 96.13 | 761358 | 38941 | 0.15 | 2.84 | contig_1 | contig_25 |
| 322291 | 322584 | 36576 | 36283 | 294 | 294 | 97.28 | 761358 | 38941 | 0.04 | 0.75 | contig_1 | contig_25 |
| 322856 | 328031 | 35981 | 30804 | 5176 | 5178 | 88.72 | 761358 | 38941 | 0.68 | 13.3 | contig_1 | contig_25 |
| 328037 | 331773 | 29948 | 26213 | 3737 | 3736 | 93.26 | 761358 | 38941 | 0.49 | 9.59 | contig_1 | contig_25 |
| 331882 | 333708 | 26199 | 24375 | 1827 | 1825 | 89.52 | 761358 | 38941 | 0.24 | 4.69 | contig_1 | contig_25 |
| 334519 | 334948 | 1874 | 1446 | 430 | 429 | 89.84 | 761358 | 66216 | 0.06 | 0.65 | contig_1 | contig_18 |
| 335406 | 335484 | 24211 | 24133 | 79 | 79 | 97.47 | 761358 | 38941 | 0.01 | 0.2 | contig_1 | contig_25 |
| 337354 | 339376 | 22593 | 20571 | 2023 | 2023 | 91.8 | 761358 | 38941 | 0.27 | 5.2 | contig_1 | contig_25 |
| 344182 | 344589 | 15115 | 14714 | 408 | 402 | 91.18 | 761358 | 38941 | 0.05 | 1.03 | contig_1 | contig_25 |
| 344830 | 345665 | 14489 | 13653 | 836 | 837 | 97.85 | 761358 | 38941 | 0.11 | 2.15 | contig_1 | contig_25 |
| 359373 | 360701 | 1333 | 1 | 1329 | 1333 | 92.28 | 761358 | 2182 | 0.17 | 61.09 | contig_1 | contig_37 |
| 360795 | 368729 | 173550 | 165619 | 7935 | 7932 | 98.19 | 761358 | 173550 | 1.04 | 4.57 | contig_1 | contig_7 |
| 371620 | 386166 | 165522 | 150941 | 14547 | 14582 | 99.04 | 761358 | 173550 | 1.91 | 8.4 | contig_1 | contig_7 |
| 386178 | 389199 | 147652 | 144631 | 3022 | 3022 | 98.64 | 761358 | 173550 | 0.4 | 1.74 | contig_1 | contig_7 |
| 394849 | 401563 | 144562 | 137844 | 6715 | 6719 | 99.4 | 761358 | 173550 | 0.88 | 3.87 | contig_1 | contig_7 |
| 401555 | 401660 | 44055 | 43952 | 106 | 104 | 96.23 | 761358 | 87176 | 0.01 | 0.12 | contig_1 | contig_38 |
| 401564 | 401696 | 1593 | 1474 | 133 | 120 | 89.47 | 761358 | 69111 | 0.02 | 0.17 | contig_1 | contig_35 |
| 402827 | 403140 | 65663 | 65350 | 314 | 314 | 98.73 | 761358 | 65669 | 0.04 | 0.48 | contig_1 | contig_11 |
| 403142 | 405237 | 2100 | 1 | 2096 | 2100 | 94.14 | 761358 | 3165 | 0.28 | 66.35 | contig_1 | contig_22 |
| 405499 | 465765 | 1 | 60345 | 60267 | 60345 | 98.67 | 761358 | 68147 | 7.92 | 88.55 | contig_1 | contig_26 |
| 407981 | 408555 | 10163 | 10739 | 575 | 577 | 79.83 | 761358 | 11334 | 0.08 | 5.09 | contig_1 | contig_28 |
| 469572 | 475431 | 60754 | 66621 | 5860 | 5868 | 98.84 | 761358 | 68147 | 0.77 | 8.61 | contig_1 | contig_26 |
| 470936 | 471020 | 102 | 186 | 85 | 85 | 100 | 761358 | 543 | 0.01 | 15.65 | contig_1 | contig_43 |
| 516442 | 516743 | 65350 | 65653 | 302 | 304 | 98.68 | 761358 | 65669 | 0.04 | 0.46 | contig_1 | contig_11 |
| 517552 | 517685 | 1474 | 1594 | 134 | 121 | 89.55 | 761358 | 69111 | 0.02 | 0.18 | contig_1 | contig_35 |
| 517588 | 517684 | 43952 | 44048 | 97 | 97 | 97.94 | 761358 | 87176 | 0.01 | 0.11 | contig_1 | contig_38 |
| 519971 | 521505 | 66608 | 68147 | 1535 | 1540 | 99.03 | 761358 | 68147 | 0.2 | 2.26 | contig_1 | contig_26 |
| 521426 | 521699 | 20872 | 20598 | 274 | 275 | 92.03 | 761358 | 97858 | 0.04 | 0.28 | contig_1 | contig_45 |
| 521506 | 522835 | 1 | 1331 | 1330 | 1331 | 98.2 | 761358 | 1479 | 0.17 | 89.99 | contig_1 | contig_34 |
| 522936 | 523306 | 1108 | 1479 | 371 | 372 | 99.19 | 761358 | 1479 | 0.05 | 25.15 | contig_1 | contig_34 |
| 523307 | 526586 | 1 | 3282 | 3280 | 3282 | 99.51 | 761358 | 33897 | 0.43 | 9.68 | contig_1 | contig_14 |
| 526592 | 550698 | 9731 | 33865 | 24107 | 24135 | 99.22 | 761358 | 33897 | 3.17 | 71.2 | contig_1 | contig_14 |
| 550707 | 559582 | 182544 | 173652 | 8876 | 8893 | 98.47 | 761358 | 183913 | 1.17 | 4.84 | contig_1 | contig_8 |
| 559718 | 574120 | 173652 | 159232 | 14403 | 14421 | 99.22 | 761358 | 183913 | 1.89 | 7.84 | contig_1 | contig_8 |
| 574108 | 610663 | 114565 | 77971 | 36556 | 36595 | 99.37 | 761358 | 183913 | 4.8 | 19.9 | contig_1 | contig_8 |
| 583419 | 585278 | 8441 | 10299 | 1860 | 1859 | 81.04 | 761358 | 74762 | 0.24 | 2.49 | contig_1 | contig_20 |
| 610666 | 612487 | 76856 | 75033 | 1822 | 1824 | 99.12 | 761358 | 183913 | 0.24 | 0.99 | contig_1 | contig_8 |
| 612470 | 650751 | 74230 | 35916 | 38282 | 38315 | 99.41 | 761358 | 183913 | 5.03 | 20.83 | contig_1 | contig_8 |
| 671970 | 707791 | 35842 | 1 | 35822 | 35842 | 99.57 | 761358 | 183913 | 4.71 | 19.49 | contig_1 | contig_8 |
| 707712 | 707976 | 20872 | 20607 | 265 | 266 | 91.42 | 761358 | 97858 | 0.03 | 0.27 | contig_1 | contig_45 |
| 707792 | 709209 | 1 | 1419 | 1418 | 1419 | 98.73 | 761358 | 1479 | 0.19 | 95.94 | contig_1 | contig_34 |
| 709367 | 709795 | 1051 | 1479 | 429 | 429 | 99.53 | 761358 | 1479 | 0.06 | 29.01 | contig_1 | contig_34 |
| 709796 | 712071 | 1 | 2279 | 2276 | 2279 | 99.03 | 761358 | 2279 | 0.3 | 100 | contig_1 | contig_42 |
| 709986 | 710063 | 45042 | 44965 | 78 | 78 | 100 | 761358 | 45043 | 0.01 | 0.17 | contig_1 | contig_44 |
| 709988 | 710063 | 187 | 262 | 76 | 76 | 100 | 761358 | 543 | 0.01 | 14 | contig_1 | contig_43 |
| 711993 | 712069 | 12 | 88 | 77 | 77 | 100 | 761358 | 543 | 0.01 | 14.18 | contig_1 | contig_43 |
| 712233 | 752415 | 1 | 40170 | 40183 | 40170 | 99.43 | 761358 | 40170 | 5.28 | 100 | contig_1 | contig_3 |
| 752577 | 753118 | 1 | 543 | 542 | 543 | 99.82 | 761358 | 543 | 0.07 | 100 | contig_1 | contig_43 |
| 752588 | 752664 | 2201 | 2277 | 77 | 77 | 100 | 761358 | 2279 | 0.01 | 3.38 | contig_1 | contig_42 |
| 752678 | 752762 | 62118 | 62202 | 85 | 85 | 100 | 761358 | 68147 | 0.01 | 0.12 | contig_1 | contig_26 |
| 752763 | 752838 | 194 | 269 | 76 | 76 | 100 | 761358 | 2279 | 0.01 | 3.33 | contig_1 | contig_42 |
| 752763 | 752838 | 45040 | 44965 | 76 | 76 | 100 | 761358 | 45043 | 0.01 | 0.17 | contig_1 | contig_44 |
| 753037 | 753115 | 4561 | 4639 | 79 | 79 | 100 | 761358 | 4729 | 0.01 | 1.67 | contig_1 | contig_2 |
| 753039 | 753193 | 44956 | 44795 | 155 | 162 | 87.04 | 761358 | 45043 | 0.02 | 0.36 | contig_1 | contig_44 |
| 753118 | 753194 | 13949 | 13873 | 77 | 77 | 100 | 761358 | 37534 | 0.01 | 0.21 | contig_1 | contig_19 |
| 753194 | 757908 | 1 | 4729 | 4715 | 4729 | 99.43 | 761358 | 4729 | 0.62 | 100 | contig_1 | contig_2 |
| 757740 | 757818 | 462 | 540 | 79 | 79 | 100 | 761358 | 543 | 0.01 | 14.55 | contig_1 | contig_43 |
| 757875 | 757983 | 44905 | 44795 | 109 | 111 | 91.89 | 761358 | 45043 | 0.01 | 0.25 | contig_1 | contig_44 |
| 757904 | 757984 | 13954 | 13873 | 81 | 82 | 98.78 | 761358 | 37534 | 0.01 | 0.22 | contig_1 | contig_19 |
| 757984 | 760761 | 1 | 2783 | 2778 | 2783 | 99.32 | 761358 | 2783 | 0.36 | 100 | contig_1 | contig_31 |
| 757988 | 758065 | 65805 | 65728 | 78 | 78 | 100 | 761358 | 65806 | 0.01 | 0.12 | contig_1 | contig_36 |
| 761222 | 761358 | 1 | 138 | 137 | 138 | 98.55 | 761358 | 530 | 0.02 | 26.04 | contig_1 | contig_41 |
| 914 | 1216 | 65669 | 65365 | 303 | 305 | 98.69 | 1216 | 65669 | 24.92 | 0.46 | contig_10 | contig_11 |
| 1 | 889 | 6 | 894 | 889 | 889 | 99.89 | 889 | 955 | 100 | 93.09 | contig_12 | contig_24 |
| 1 | 129 | 1589 | 1474 | 129 | 116 | 89.15 | 850 | 69111 | 15.18 | 0.17 | contig_13 | contig_35 |
| 1 | 93 | 44044 | 43952 | 93 | 93 | 97.85 | 850 | 87176 | 10.94 | 0.11 | contig_13 | contig_38 |
| 261 | 583 | 65343 | 65669 | 323 | 327 | 98.17 | 610 | 65669 | 52.95 | 0.5 | contig_15 | contig_11 |
| 525 | 22074 | 74458 | 52876 | 21550 | 21583 | 99.33 | 743670 | 74458 | 2.9 | 28.99 | contig_2 | contig_23 |
| 527 | 657 | 2781 | 2651 | 131 | 131 | 93.13 | 743670 | 2783 | 0.02 | 4.71 | contig_2 | contig_31 |
| 22211 | 54420 | 52742 | 20498 | 32210 | 32245 | 98.95 | 743670 | 74458 | 4.33 | 43.31 | contig_2 | contig_23 |
| 54652 | 75404 | 20841 | 74 | 20753 | 20768 | 98.18 | 743670 | 74458 | 2.79 | 27.89 | contig_2 | contig_23 |
| 76517 | 95570 | 1 | 19078 | 19054 | 19078 | 99 | 743670 | 65806 | 2.56 | 28.99 | contig_2 | contig_36 |
| 95569 | 113724 | 19288 | 37438 | 18156 | 18151 | 99.14 | 743670 | 65806 | 2.44 | 27.58 | contig_2 | contig_36 |
| 113813 | 123191 | 38933 | 48335 | 9379 | 9403 | 98.97 | 743670 | 65806 | 1.26 | 14.29 | contig_2 | contig_36 |
| 125051 | 138628 | 52222 | 65806 | 13578 | 13585 | 99.39 | 743670 | 65806 | 1.83 | 20.64 | contig_2 | contig_36 |
| 138550 | 138627 | 82 | 5 | 78 | 78 | 100 | 743670 | 2783 | 0.01 | 2.8 | contig_2 | contig_31 |
| 138704 | 163455 | 1 | 24777 | 24752 | 24777 | 99.03 | 743670 | 142918 | 3.33 | 17.34 | contig_2 | contig_39 |
| 164029 | 164192 | 24778 | 24942 | 164 | 165 | 93.98 | 743670 | 142918 | 0.02 | 0.12 | contig_2 | contig_39 |
| 164195 | 177796 | 28093 | 41701 | 13602 | 13609 | 99.13 | 743670 | 142918 | 1.83 | 9.52 | contig_2 | contig_39 |
| 178978 | 241068 | 41695 | 103808 | 62091 | 62114 | 99.13 | 743670 | 142918 | 8.35 | 43.46 | contig_2 | contig_39 |
| 246714 | 263983 | 103800 | 121110 | 17270 | 17311 | 97.54 | 743670 | 142918 | 2.32 | 12.11 | contig_2 | contig_39 |
| 264799 | 284380 | 121111 | 140725 | 19582 | 19615 | 98.5 | 743670 | 142918 | 2.63 | 13.72 | contig_2 | contig_39 |
| 275940 | 276041 | 71753 | 71857 | 102 | 105 | 93.33 | 743670 | 173550 | 0.01 | 0.06 | contig_2 | contig_7 |
| 432321 | 432627 | 2763 | 2463 | 307 | 301 | 91.21 | 743670 | 66216 | 0.04 | 0.45 | contig_2 | contig_18 |
| 465541 | 467192 | 140716 | 142369 | 1652 | 1654 | 99.21 | 743670 | 142918 | 0.22 | 1.16 | contig_2 | contig_39 |
| 473724 | 540656 | 161100 | 94077 | 66933 | 67024 | 98.7 | 743670 | 162498 | 9 | 41.25 | contig_2 | contig_32 |
| 542996 | 543093 | 43952 | 44048 | 98 | 97 | 97.96 | 743670 | 87176 | 0.01 | 0.11 | contig_2 | contig_38 |
| 552005 | 564606 | 92370 | 79781 | 12602 | 12590 | 98.76 | 743670 | 162498 | 1.69 | 7.75 | contig_2 | contig_32 |
| 564903 | 570877 | 79747 | 73830 | 5975 | 5918 | 94.15 | 743670 | 162498 | 0.8 | 3.64 | contig_2 | contig_32 |
| 570910 | 573989 | 73863 | 70785 | 3080 | 3079 | 96.88 | 743670 | 162498 | 0.41 | 1.89 | contig_2 | contig_32 |
| 574391 | 594428 | 70392 | 50376 | 20038 | 20017 | 96.13 | 743670 | 162498 | 2.69 | 12.32 | contig_2 | contig_32 |
| 594427 | 594749 | 65348 | 65669 | 323 | 322 | 99.07 | 743670 | 65669 | 0.04 | 0.49 | contig_2 | contig_11 |
| 595615 | 595747 | 1474 | 1593 | 133 | 120 | 89.47 | 743670 | 69111 | 0.02 | 0.17 | contig_2 | contig_35 |
| 595651 | 595753 | 43952 | 44054 | 103 | 103 | 97.09 | 743670 | 87176 | 0.01 | 0.12 | contig_2 | contig_38 |
| 595749 | 623979 | 50382 | 22107 | 28231 | 28276 | 98.87 | 743670 | 162498 | 3.8 | 17.4 | contig_2 | contig_32 |
| 623983 | 624302 | 65350 | 65669 | 320 | 320 | 98.14 | 743670 | 65669 | 0.04 | 0.49 | contig_2 | contig_11 |
| 625004 | 625136 | 1474 | 1593 | 133 | 120 | 89.47 | 743670 | 69111 | 0.02 | 0.17 | contig_2 | contig_35 |
| 625040 | 625137 | 43952 | 44049 | 98 | 98 | 97.96 | 743670 | 87176 | 0.01 | 0.11 | contig_2 | contig_38 |
| 625137 | 645369 | 22111 | 1847 | 20233 | 20265 | 98.9 | 743670 | 162498 | 2.72 | 12.47 | contig_2 | contig_32 |
| 645925 | 647780 | 1848 | 1 | 1856 | 1848 | 98.71 | 743670 | 162498 | 0.25 | 1.14 | contig_2 | contig_32 |
| 647544 | 647778 | 237 | 3 | 235 | 235 | 94.89 | 743670 | 18586 | 0.03 | 1.26 | contig_2 | contig_15 |
| 647781 | 648208 | 955 | 518 | 428 | 438 | 97.72 | 743670 | 955 | 0.06 | 45.86 | contig_2 | contig_24 |
| 648699 | 649017 | 321 | 2 | 319 | 320 | 99.38 | 743670 | 955 | 0.04 | 33.51 | contig_2 | contig_24 |
| 649018 | 699665 | 1 | 50691 | 50648 | 50691 | 99.33 | 743670 | 91667 | 6.81 | 55.3 | contig_2 | contig_4 |
| 699666 | 699985 | 65350 | 65669 | 320 | 320 | 99.38 | 743670 | 65669 | 0.04 | 0.49 | contig_2 | contig_11 |
| 701366 | 701506 | 1474 | 1600 | 141 | 127 | 88.65 | 743670 | 69111 | 0.02 | 0.18 | contig_2 | contig_35 |
| 701402 | 701498 | 43952 | 44048 | 97 | 97 | 97.94 | 743670 | 87176 | 0.01 | 0.11 | contig_2 | contig_38 |
| 701499 | 702253 | 50684 | 51437 | 755 | 754 | 98.15 | 743670 | 91667 | 0.1 | 0.82 | contig_2 | contig_4 |
| 702253 | 705158 | 52760 | 55669 | 2906 | 2910 | 99.14 | 743670 | 91667 | 0.39 | 3.17 | contig_2 | contig_4 |
| 705477 | 712107 | 55685 | 62317 | 6631 | 6633 | 99.08 | 743670 | 91667 | 0.89 | 7.24 | contig_2 | contig_4 |
| 712474 | 734772 | 62320 | 84640 | 22299 | 22321 | 99.16 | 743670 | 91667 | 3 | 24.35 | contig_2 | contig_4 |
| 735814 | 736452 | 84818 | 85444 | 639 | 627 | 95.62 | 743670 | 91667 | 0.09 | 0.68 | contig_2 | contig_4 |
| 736650 | 739669 | 85440 | 88459 | 3020 | 3020 | 98.51 | 743670 | 91667 | 0.41 | 3.29 | contig_2 | contig_4 |
| 739860 | 740369 | 88463 | 88971 | 510 | 509 | 97.25 | 743670 | 91667 | 0.07 | 0.56 | contig_2 | contig_4 |
| 740607 | 743315 | 88958 | 91667 | 2709 | 2710 | 99.52 | 743670 | 91667 | 0.36 | 2.96 | contig_2 | contig_4 |
| 743317 | 743670 | 1 | 358 | 354 | 358 | 98.6 | 743670 | 3211 | 0.05 | 11.15 | contig_2 | contig_40 |
| 1 | 42924 | 889 | 43863 | 42924 | 42975 | 99.17 | 483988 | 65669 | 8.87 | 65.44 | contig_3 | contig_11 |
| 1 | 249 | 59927 | 60175 | 249 | 249 | 85.94 | 483988 | 97858 | 0.05 | 0.25 | contig_3 | contig_45 |
| 3313 | 3520 | 37554 | 37344 | 208 | 211 | 79.82 | 483988 | 38220 | 0.04 | 0.55 | contig_3 | contig_27 |
| 42924 | 43243 | 65349 | 65669 | 320 | 321 | 99.07 | 483988 | 65669 | 0.07 | 0.49 | contig_3 | contig_11 |
| 44259 | 44393 | 1474 | 1595 | 135 | 122 | 89.63 | 483988 | 69111 | 0.03 | 0.18 | contig_3 | contig_35 |
| 44295 | 44391 | 43952 | 44048 | 97 | 97 | 97.94 | 483988 | 87176 | 0.02 | 0.11 | contig_3 | contig_38 |
| 44392 | 66177 | 43856 | 65669 | 21786 | 21814 | 99 | 483988 | 65669 | 4.5 | 33.22 | contig_3 | contig_11 |
| 58604 | 58734 | 79635 | 79505 | 131 | 131 | 94.66 | 483988 | 85366 | 0.03 | 0.15 | contig_3 | contig_33 |
| 67191 | 87037 | 1474 | 21320 | 19847 | 19847 | 98.9 | 483988 | 69111 | 4.1 | 28.72 | contig_3 | contig_35 |
| 91825 | 101807 | 28195 | 38181 | 9983 | 9987 | 98.83 | 483988 | 69111 | 2.06 | 14.45 | contig_3 | contig_35 |
| 102755 | 123381 | 38178 | 58834 | 20627 | 20657 | 98.57 | 483988 | 69111 | 4.26 | 29.89 | contig_3 | contig_35 |
| 123654 | 131607 | 59098 | 67055 | 7954 | 7958 | 98.03 | 483988 | 69111 | 1.64 | 11.51 | contig_3 | contig_35 |
| 143895 | 148709 | 6526 | 11334 | 4815 | 4809 | 94.09 | 483988 | 11334 | 0.99 | 42.43 | contig_3 | contig_28 |
| 148294 | 148476 | 1477 | 1660 | 183 | 184 | 87.03 | 483988 | 68147 | 0.04 | 0.27 | contig_3 | contig_26 |
| 148690 | 148769 | 1512 | 1591 | 80 | 80 | 100 | 483988 | 69111 | 0.02 | 0.12 | contig_3 | contig_35 |
| 148772 | 166590 | 23842 | 6017 | 17819 | 17826 | 99.18 | 483988 | 23845 | 3.68 | 74.76 | contig_3 | contig_5 |
| 170451 | 175050 | 4601 | 1 | 4600 | 4601 | 99 | 483988 | 23845 | 0.95 | 19.3 | contig_3 | contig_5 |
| 175222 | 220215 | 45043 | 1 | 44994 | 45043 | 98.67 | 483988 | 45043 | 9.3 | 100 | contig_3 | contig_44 |
| 175223 | 175300 | 192 | 269 | 78 | 78 | 100 | 483988 | 2279 | 0.02 | 3.42 | contig_3 | contig_42 |
| 175225 | 175300 | 187 | 262 | 76 | 76 | 100 | 483988 | 543 | 0.02 | 14 | contig_3 | contig_43 |
| 175395 | 175470 | 13949 | 13874 | 76 | 76 | 100 | 483988 | 37534 | 0.02 | 0.2 | contig_3 | contig_19 |
| 220208 | 223422 | 3 | 3220 | 3215 | 3218 | 98.82 | 483988 | 10995 | 0.66 | 29.27 | contig_3 | contig_10 |
| 221766 | 222832 | 2511 | 3577 | 1067 | 1067 | 84.77 | 483988 | 10995 | 0.22 | 9.7 | contig_3 | contig_10 |
| 223536 | 224793 | 1561 | 2831 | 1258 | 1271 | 84.04 | 483988 | 10995 | 0.26 | 11.56 | contig_3 | contig_10 |
| 223538 | 232005 | 2511 | 10995 | 8468 | 8485 | 96.29 | 483988 | 10995 | 1.75 | 77.17 | contig_3 | contig_10 |
| 226226 | 232025 | 5209 | 10995 | 5800 | 5787 | 98.21 | 483988 | 10995 | 1.2 | 52.63 | contig_3 | contig_10 |
| 231914 | 232004 | 1 | 91 | 91 | 91 | 100 | 483988 | 97858 | 0.02 | 0.09 | contig_3 | contig_45 |
| 231994 | 232085 | 10904 | 10995 | 92 | 92 | 100 | 483988 | 10995 | 0.02 | 0.84 | contig_3 | contig_10 |
| 232014 | 234799 | 1 | 2787 | 2786 | 2787 | 98.49 | 483988 | 97858 | 0.58 | 2.85 | contig_3 | contig_45 |
| 235006 | 236044 | 2994 | 4037 | 1039 | 1044 | 96.84 | 483988 | 97858 | 0.21 | 1.07 | contig_3 | contig_45 |
| 236036 | 281572 | 11993 | 57571 | 45537 | 45579 | 98.7 | 483988 | 97858 | 9.41 | 46.58 | contig_3 | contig_45 |
| 244631 | 244818 | 195 | 5 | 188 | 191 | 90.1 | 483988 | 1479 | 0.04 | 12.91 | contig_3 | contig_34 |
| 281736 | 308497 | 62474 | 89257 | 26762 | 26784 | 99.07 | 483988 | 97858 | 5.53 | 27.37 | contig_3 | contig_45 |
| 319943 | 328410 | 89369 | 97858 | 8468 | 8490 | 98.45 | 483988 | 97858 | 1.75 | 8.68 | contig_3 | contig_45 |
| 352560 | 352789 | 65349 | 65580 | 230 | 232 | 97 | 483988 | 65669 | 0.05 | 0.35 | contig_3 | contig_11 |
| 354138 | 354268 | 1474 | 1593 | 131 | 120 | 89.39 | 483988 | 69111 | 0.03 | 0.17 | contig_3 | contig_35 |
| 354175 | 354268 | 43955 | 44048 | 94 | 94 | 97.87 | 483988 | 87176 | 0.02 | 0.11 | contig_3 | contig_38 |
| 354629 | 409314 | 66092 | 11387 | 54686 | 54706 | 98.82 | 483988 | 66216 | 11.3 | 82.62 | contig_3 | contig_18 |
| 409134 | 483549 | 299 | 74762 | 74416 | 74464 | 99.19 | 483988 | 74762 | 15.38 | 99.6 | contig_3 | contig_20 |
| 417266 | 418212 | 105239 | 104293 | 947 | 947 | 82.53 | 483988 | 183913 | 0.2 | 0.51 | contig_3 | contig_8 |
| 483639 | 483988 | 1 | 351 | 350 | 351 | 99.15 | 483988 | 3211 | 0.07 | 10.93 | contig_3 | contig_40 |
| 191 | 326 | 1474 | 1596 | 136 | 123 | 89.71 | 117003 | 69111 | 0.12 | 0.18 | contig_4 | contig_35 |
| 207 | 331 | 59755 | 59879 | 125 | 125 | 90.4 | 117003 | 69111 | 0.11 | 0.18 | contig_4 | contig_35 |
| 227 | 323 | 43952 | 44048 | 97 | 97 | 97.94 | 117003 | 87176 | 0.08 | 0.11 | contig_4 | contig_38 |
| 356 | 18919 | 18586 | 1 | 18564 | 18586 | 99.38 | 117003 | 18586 | 15.87 | 100 | contig_4 | contig_15 |
| 18683 | 18917 | 237 | 3 | 235 | 235 | 95.32 | 117003 | 162498 | 0.2 | 0.14 | contig_4 | contig_32 |
| 18920 | 19435 | 955 | 439 | 516 | 517 | 99.61 | 117003 | 955 | 0.44 | 54.14 | contig_4 | contig_24 |
| 19724 | 20038 | 315 | 1 | 315 | 315 | 99.68 | 117003 | 955 | 0.27 | 32.98 | contig_4 | contig_24 |
| 20039 | 58374 | 85366 | 46986 | 38336 | 38381 | 99.52 | 117003 | 85366 | 32.76 | 44.96 | contig_4 | contig_33 |
| 25762 | 25892 | 58078 | 58208 | 131 | 131 | 94.66 | 117003 | 65669 | 0.11 | 0.2 | contig_4 | contig_11 |
| 58509 | 76776 | 41555 | 23271 | 18268 | 18285 | 99.37 | 117003 | 85366 | 15.61 | 21.42 | contig_4 | contig_33 |
| 76757 | 98561 | 45146 | 23344 | 21805 | 21803 | 99.05 | 117003 | 56789 | 18.64 | 38.39 | contig_4 | contig_6 |
| 98881 | 116704 | 17849 | 2 | 17824 | 17848 | 99.16 | 117003 | 56789 | 15.23 | 31.43 | contig_4 | contig_6 |
| 116705 | 117003 | 1 | 299 | 299 | 299 | 100 | 117003 | 3211 | 0.26 | 9.31 | contig_4 | contig_40 |
| 1 | 279 | 279 | 1 | 279 | 279 | 98.93 | 102317 | 1596 | 0.27 | 17.48 | contig_5 | contig_16 |
| 406 | 33567 | 53256 | 20070 | 33162 | 33187 | 99.36 | 102317 | 53256 | 32.41 | 62.32 | contig_5 | contig_9 |
| 33548 | 36320 | 19969 | 17198 | 2773 | 2772 | 95.71 | 102317 | 53256 | 2.71 | 5.21 | contig_5 | contig_9 |
| 36247 | 36357 | 17421 | 17309 | 111 | 113 | 96.46 | 102317 | 53256 | 0.11 | 0.21 | contig_5 | contig_9 |
| 36757 | 37062 | 17421 | 17115 | 306 | 307 | 94.14 | 102317 | 53256 | 0.3 | 0.58 | contig_5 | contig_9 |
| 39096 | 55194 | 16240 | 60 | 16099 | 16181 | 97.7 | 102317 | 53256 | 15.73 | 30.38 | contig_5 | contig_9 |
| 60409 | 101942 | 4 | 41587 | 41534 | 41584 | 99.36 | 102317 | 41587 | 40.59 | 99.99 | contig_5 | contig_17 |
| 101943 | 102317 | 1 | 374 | 375 | 374 | 99.2 | 102317 | 3211 | 0.37 | 11.65 | contig_5 | contig_40 |
| 258 | 786 | 1 | 529 | 529 | 529 | 98.68 | 43902 | 530 | 1.2 | 99.81 | contig_6 | contig_41 |
| 265 | 787 | 7 | 532 | 523 | 526 | 83.3 | 43902 | 532 | 1.19 | 98.87 | contig_6 | contig_12 |
| 407 | 2115 | 58464 | 60175 | 1709 | 1712 | 83.41 | 43902 | 97858 | 3.89 | 1.75 | contig_6 | contig_45 |
| 979 | 2217 | 1 | 1239 | 1239 | 1239 | 92.1 | 43902 | 65669 | 2.82 | 1.89 | contig_6 | contig_11 |
| 2541 | 3111 | 2413 | 2983 | 571 | 571 | 86.89 | 43902 | 65669 | 1.3 | 0.87 | contig_6 | contig_11 |
| 3560 | 3848 | 3315 | 3603 | 289 | 289 | 88.93 | 43902 | 65669 | 0.66 | 0.44 | contig_6 | contig_11 |
| 4089 | 4390 | 3861 | 4163 | 302 | 303 | 87.95 | 43902 | 65669 | 0.69 | 0.46 | contig_6 | contig_11 |
| 4578 | 11500 | 37571 | 30638 | 6923 | 6934 | 97.22 | 43902 | 38220 | 15.77 | 18.14 | contig_6 | contig_27 |
| 4595 | 4805 | 4201 | 4409 | 211 | 209 | 79.26 | 43902 | 65669 | 0.48 | 0.32 | contig_6 | contig_11 |
| 4766 | 4901 | 62171 | 62303 | 136 | 133 | 87.77 | 43902 | 97858 | 0.31 | 0.14 | contig_6 | contig_45 |
| 11518 | 12348 | 9588 | 10418 | 831 | 831 | 83.15 | 43902 | 11334 | 1.89 | 7.33 | contig_6 | contig_28 |
| 13165 | 41472 | 28349 | 1 | 28308 | 28349 | 98.89 | 43902 | 38220 | 64.48 | 74.17 | contig_6 | contig_27 |
| 41548 | 43518 | 1977 | 1 | 1971 | 1977 | 99.39 | 43902 | 1977 | 4.49 | 100 | contig_6 | contig_30 |
| 43545 | 43902 | 1 | 360 | 358 | 360 | 99.17 | 43902 | 1596 | 0.82 | 22.56 | contig_6 | contig_16 |
| 1 | 254 | 254 | 1 | 254 | 254 | 100 | 38361 | 1596 | 0.66 | 15.91 | contig_7 | contig_16 |
| 342 | 37813 | 1 | 37534 | 37472 | 37534 | 99.3 | 38361 | 37534 | 97.68 | 100 | contig_7 | contig_19 |
| 14201 | 14276 | 44795 | 44870 | 76 | 76 | 100 | 38361 | 45043 | 0.2 | 0.17 | contig_7 | contig_44 |
| 37904 | 38361 | 1 | 458 | 458 | 458 | 98.91 | 38361 | 3211 | 1.19 | 14.26 | contig_7 | contig_40 |
| 1 | 364 | 365 | 1 | 364 | 365 | 99.73 | 10817 | 1596 | 3.37 | 22.87 | contig_8 | contig_16 |
| 433 | 10461 | 10042 | 1 | 10029 | 10042 | 99.38 | 10817 | 10043 | 92.72 | 99.99 | contig_8 | contig_21 |
| 10463 | 10817 | 1 | 354 | 355 | 354 | 99.44 | 10817 | 3211 | 3.28 | 11.02 | contig_8 | contig_40 |
| 6 | 3216 | 1 | 3211 | 3211 | 3211 | 99.72 | 5854 | 3211 | 54.85 | 100 | contig_9 | contig_40 |
| 3238 | 3657 | 1596 | 1178 | 420 | 419 | 99.52 | 5854 | 1596 | 7.17 | 26.25 | contig_9 | contig_16 |
| 3800 | 3881 | 14038 | 13957 | 82 | 82 | 98.78 | 5854 | 37534 | 1.4 | 0.22 | contig_9 | contig_19 |
| 3973 | 5565 | 1593 | 1 | 1593 | 1593 | 99.94 | 5854 | 1596 | 27.21 | 99.81 | contig_9 | contig_16 |
| 5671 | 5854 | 65350 | 65542 | 184 | 193 | 94.82 | 5854 | 65669 | 3.14 | 0.29 | contig_9 | contig_11 |
